# Influence of microRNAs and Long Non-Coding RNAs in Cancer Chemoresistance

**DOI:** 10.3390/genes8030095

**Published:** 2017-03-03

**Authors:** Duncan Ayers, Jo Vandesompele

**Affiliations:** 1 Centre for Molecular Medicine and Biobanking, University of Malta, Msida MSD2080, Malta; 2 Faculty of Biology, Medicine and Health, The University of Manchester, Manchester M1 7DN, UK; 3 Center for Medical Genetics Ghent, Ghent University, Ghent 9000, Belgium; Joke.Vandesompele@UGent.be; 4 Cancer Research Institute Ghent (CRIG), Ghent University, Ghent 9000, Belgium

**Keywords:** miRNA, lncRNA, cancer, chemoresistance, drug resistance, tumour, microRNA

## Abstract

Innate and acquired chemoresistance exhibited by most tumours exposed to conventional chemotherapeutic agents account for the majority of relapse cases in cancer patients. Such chemoresistance phenotypes are of a multi-factorial nature from multiple key molecular players. The discovery of the RNA interference pathway in 1998 and the widespread gene regulatory influences exerted by microRNAs (miRNAs) and other non-coding RNAs have certainly expanded the level of intricacy present for the development of any single physiological phenotype, including cancer chemoresistance. This review article focuses on the latest research efforts in identifying and validating specific key molecular players from the two main families of non-coding RNAs, namely miRNAs and long non-coding RNAs (lncRNAs), having direct or indirect influences in the development of cancer drug resistance properties and how such knowledge can be utilised for novel theranostics in oncology.

## 1. Introduction

The discovery of the RNA interference pathway in 1998 and the widespread gene regulatory influences exerted by microRNAs (miRNAs) and other non-coding RNAs (ncRNAs) have certainly expanded the level of intricacy present for the development of any single physiological phenotype [1]. Such phenotypes can include clinical conditions of either an acute or a chronic nature. Undoubtedly, cancer best symbolizes clinical conditions relying on multifactorial influences for its development. Furthermore, the clinical presentation of any specific cancer condition in the patient can vary to great extents, depending on multiple tumour characteristics such as the degree of invasiveness, aggressiveness and angiogenesis. However, one of the most crucial cancer phenotypes that pose a major challenge to current conventional chemotherapeutic measures is the ability of the tumour to withstand the pharmacological effects of multiple cancer chemotherapy drugs, typically described as chemoresistance.

Since the influences of ncRNAs in the main facets of cancer development are described in great detail within the scientific literature, this review specifically places a spotlight on the emerging global research efforts that (in the authors’ opinion) most effectively recognize the growing link pertaining to non-coding RNA activities with the regulation of cancer chemoresistance properties [2,3,4,5,6].

## 2. Cancer Chemoresistance Manifestations and Development Mechanisms

Tumours bearing a chemoresistance phenotype can irrevocably thwart the prognosis of the cancer patient, particularly when such characteristics evolve in relapse of the disease. This chemoresistance phenotype has two distinct development mechanisms, leading to the existence of innate and acquired chemoresistance phenotypes [7].

Innate chemoresistance refers to the scenario that an individual tumour can inherently possess unique genetic characteristics that render the tumour to withstand single (or multiple) chemotherapeutic agents, through various influences on drug cytotoxicity circumvention pathways, as described below [7].

In the case of acquired chemoresistance properties, the link between such cancer relapse phases and multi-drug resistant (MDR) tumours is predominantly due to the regular exposure of the tumour to conventional chemotherapeutic cyclical administration [7]. This essentially drives the tumour to evolve at the genetic level to a variant having increased withstanding potential against such conventional chemotherapeutic agents [7].

Since this article focuses specifically on the links identified so far between miRNAs/lncRNAs and cancer chemoresistance properties, it is important to highlight the main recognized mechanisms by which tumours can develop multi-drug resistance against conventional chemotherapeutic agents.

By far the most important and characterised mechanism for the emergence of cancer chemoresistance properties is the employment of drug efflux pumps that actively remove multiple drugs from the tumour cell cytoplasm, including conventional chemotherapeutic agents and eventually leading to MDR [8,9,10,11,12]. The key molecular players involved in drug efflux processes are primarily the ATP-dependent binding cassette (ABC) transporters such as ABCG2, ABCB1 (multidrug resistance 1 gene/P-glycoprotein) and ABCC1 (multidrug resistance-associated protein 1) [8,9,10,11,12]. Another important mechanistic branch leading to chemoresistance is the dysfunction or loss of p53-mediated apoptotic pathways typically triggered by DNA damage, with examples being dysfunctional activity of the mouse double minute 2 gene (Mdm2) and the p53 encoding gene (TP53) [13,14,15]. In a similar manner, other pro-apoptotic pathways that are typically triggered by cytotoxic drug activities can be hindered within chemoresistant tumour cells. Such pathway issues include cellular FADD-like interleukin 1 beta converting enzyme-inhibitory protein (c-FLIP) and the Bcl-2 protein family members [16,17,18,19]. Alternatively, triggering of proliferative/survival signalling pathways such as the ERK and PI3K pathways by means of protein tyrosine kinases, sirtulins, transcription factor kappa B (NFκB) or epidermal growth factor receptor (EGFR) family members can also lead to chemoresistance phenotypes within tumours [20,21,22]. Furthermore, increased efforts by key molecular components of the nucleotide excision repair pathway can take place as a means of limiting tumour cell DNA damage by cytotoxic drug activity [23,24,25]. Other mechanisms directing chemoresistance phenotypes in tumours include drug modulation through inactivation or attenuation of cytotoxic drug activity, modification of drug targets and inhibition of tumour suppressor genes that trigger DNA methylation pathways [26,27,28].

In essence, a particular tumour can be clinically recognized as MDR through the identification of a unique spectrum of dysregulated expression patterns of multiple mRNA biomarkers [29,30,31,32,33]. However, the discovery of the miRNA and long non-coding RNA (lncRNA) families have created additional layers of genomic regulatory functions [2,34,35,36,37,38,39]. Further insight into the roles conducted by miRNAs and lncRNAs in the development of innate and/or acquired chemoresistance properties by tumours is expected to lead to a more accurate depiction of the cancer patient’s condition—both at diagnosis and during possible relapse condition.

The sections below represent a comprehensive summary of the global research efforts in delineating the influences and main mechanistic links of such non-coding RNA families on this specific tumour characteristic. Ultimately, the goal of developing novel theranostic measures for employment in the oncology clinic setting can be attained.

## 3. Influences of miRNAs in Cancer Chemoresistance

Each physiologically active, individual miRNA consists of a 19–22 nucleotide RNA duplex, bearing a guide strand that is fully or partially complementary to the target transcript to which it binds [40]. This binding, which is typically non-totally complementary, leads to either mRNA target cleavage or a hindering effect on the translational phase of protein synthesis by the ribosomal infrastructure. This has the ultimate effect of a reduction in protein level production for the affected target transcript, or effectively post-transcriptional gene regulation [40].

Since the discovery of the initial concept of miRNA-driven gene regulation in living organisms at the turn of the millennium, close to 2600 miRNAs have been identified and catalogued in human [41]. This large family of gene regulating molecular players leads to a myriad of possibilities to the degree of beneficial and detrimental physiological interactions within the cellular microenvironment, including cancer chemoresistance properties.

Table 1 and Supplementary Materials Table S1 comprise an exhaustive compendium of studies directly focusing on the influence, through dysregulated expression, of specific miRNAs on cancer chemoresistance in the past three years alone:

The list of scientific literature depicting the involvement of miRNAs in cancer chemoresistance, as described above, highlights specific trends that require chemoresistance biomarker investigators to delve further for enhanced insight.

In essence, over 10% of all reported research findings of miRNA influences in cancer chemoresistance demonstrated that such a phenotype modulation was effected through simultaneous dysregulation of multiple miRNAs, rather than an individual putative chemoresistance miRNA—though no specific miRNA combination was identified as most prevalent by the authors [44,46,47,48,49,62,67,71,76,81,85,88,132,133,158,168,201,203,211,212,213,217,223,224,228,230]. This suggests that miRNA influences on cellular functions also occur at a more complex level, whereby it is a signature dysregulated expression pattern of two (or more) miRNAs that trigger simultaneous downregulation of a specific set of target transcripts leading to an ultimate change in cellular/tissue phenotype. Further evidence for this can be concluded by previous efforts carried out by the authors, whereby a set of seven putative chemoresistance miRNAs were identified for neuroblastoma—a paediatric cancer model [232].

The mechanistic links between miRNA activity and chemoresistance development that have been identified so far reveal that miRNA dysregulated expression can indeed influence the main cellular pathways (described in Section 2 above) that are directly affecting chemoresistance emergence in tumour models.

In the first instance, miRNAs have been recognized to regulate MDR-related molecular players such as multidrug resistance-associated protein 1 (MRP-1) [66]. The study carried out by Gao and colleagues identified the gene regulatory effects of miR-145-5p on MRP-1, ultimately enhancing the level of chemosensitivity in the doxorubicin resistant MCF-7 breast cancer cell line [66]. Furthermore, the study performed by Zhan and colleagues identified that MRP-1 could also be downregulated through the overexpression of miR-145-5p in cisplatin-resistant gallbladder tumour models, resulting in sensitizing of the tumour cells to cisplatin activity [99]. Similarly, the study conducted by Zhao and colleagues revealed that a combination of four miRNAs, namely miR-302a-3p/b-3p/c-3p/d-3p, concomitantly provide similar effects in doxorubicin-resistant breast cancer MCF-7 cell lines [71]. This study confirmed through RT-qPCR and Western blotting techniques that over-expression of these miRNAs induced sensitization to doxorubicin through a reduction in the expression level of MAP/ERK kinase 1 (MEKK1), leading to an overall downregulation of P-glycoprotein (P-gp) expression [71].

Other studies have demonstrated miRNA influences on the p53 pathway status as a means of inducing chemoresistance properties. The study conducted by Shen and colleagues recognized the effect of up-regulated miR-29a-3p in exacerbating doxorubicin-resistance in breast cancer cell lines, through its regulatory function on PTEN and GSK3β, that are two major components of the PTEN/AKT/GSK3β signalling pathway providing feedback to TP53 [55]. Further evidence for such miRNA influences on p53 modulation include the study carried out by Qin and colleagues, who revealed that the over-expression of miR-182-5p can induce cisplatin chemoresistance in hepatocellular carcinoma HepG2-Rcells [130]. This exacerbation was found to occur through the direct regulatory effect of the miRNA on tumour protein 53-induced nuclear protein 1 (TP53INP1) [130].

Evidence for the mechanistic link between miRNA activity and apoptotic pathway dysfunction for the emergence of chemoresistance in cancer includes the study performed by Zhang and colleagues [89]. The outcome of this investigation identified the exacerbating effect of miR-425-5p on chemoresistance in colorectal cancer HCT116 cell lines as well as in xenograft models, through direct action on programmed cell death 10 (PCD10) [89]. In addition, Stojcheva and colleagues reported the acquisition of chemoresistance properties by glioblastoma to alkylators such as temozolomide due to miR-138-5p direct regulatory function on BIM, which is a Bcl-2 interacting mediator of apoptosis [111]. Furthermore, miR-182-5p was also identified as playing a key role in the development of chemoresistance in ovarian carcinomas due to its gene-regulating capacity on programmed cell death 4 (PDCD4) [214].

The influences of miRNA activity on cell proliferative function, as another mechanism for chemoresistance emergence, is described through the study performed by Ye and colleagues on chemoresistant breast cancer cell line models [59]. This seminal investigation recognized the downregulatory effect of miR-484 on cytidine deaminase (CDA), which is a major molecular player in controlling cell proliferation through its suppressive function on cyclin E-CDK2 signalling, thereby inhibiting any cell-cycle progress [59]. Interestingly, the investigation conducted by Phatak and colleagues on oesophageal squamous cancer cell line models revealed that overexpression of miR-214-3p resulted in the sensitization of such tissue cultures to cisplatin [171]. Such a reduction in chemoresistance by miR-214-3p was mediated by direct regulation of surviving expression, together with indirect regulation by means of downregulation of CUG-BP1, leading to decreased mRNA stability in the targeted tumour cells [171].

Cytotoxic drug-induced DNA damage response pathway was also found to be affected by miRNA influence. The study performed by Li and colleagues identified that such a pathway can be activated by upregulation of the inhibitor of growth 5 (ING5) gene, which is in turn regulated by miR-193a-3p within the bladder cancer cell line 5637 [49]. In addition, inhibition of ING5 in this study resulted in a drastic reduction of the DNA damage response pathway within the same cell line [49].

Evidence for the modulation of cytotoxic drug targets by miRNAs can be reflected in the study performed by Liang and colleagues on gemcitabine resistant MiaPiaCa-2 pancreatic cell cancer models [216]. Results of this study led to the regulatory role of miR-33a in β-catenin downregulation, with the latter being a key molecular player in directing the expression levels of multiple genes including cyclin D1, surviving and MDR-1 [216].

In addition to the effect of multiple miRNAs on cancer chemoresistance, several studies reported the possible influences exhibited by circulating miRNAs (in blood), typically through extracellular vesicle or exosome transport [67,168,172,205,228].

Exosomes can be characterized as endocytic vesicles that can be secreted by multiple cell types within the human body, carrying a spectrum of molecular players, including miRNAs, for the purpose of cell-cell communication (e.g., antigen presentation) [233]. The transfer of genomic components such as mRNA and miRNAs between two individual cells of varying morphology, such as from bone marrow to mast cells, was also identified to lead to novel protein synthesis within the recipient cells [233]. Consequently, exosomal transfer can be reliably considered as an additional method of influence on multiple molecular pathways, since the desired physiological effect/s is induced from cellular populations situation within remote locations.

The effect of exosome activity can also be linked within the context of cancer chemoresistance, such as the findings described by Chen and colleagues [67]. This study highlighted the effect of exosomes on chemoresistant breast cancer cell lines, focusing on the transfer of miR-222-3p from doxorubicin resistant MCF-7 cell line to a chemosensitive MCF-7 cell line model [67]. This exosomal transfer ultimately rendered the recipient breast carcinoma cell line more resistant to doxorubicin activity following post-transfer functional assays [67].

Furthermore, the study carried out by Au Yeung and colleagues identified and validated the effect of exosomal transfer of miRNAs in drug resistant ovarian cancer models [205]. This study identified the exosomal transfer of miR-21-5p from cancer-associated adipocytes (within the omental stroma) into ovarian carcinoma cell populations [205]. This transfer ultimately conferred paclitaxel chemoresistance properties to the recipient ovarian carcinoma cells due to the direct regulatory effect of miR-21-5p on the transcript for apoptotic protease activating factor 1 (APAF1) [205].

Exosome transfer was also identified as being implicated in chemoresistance conferring to prostate cancer, as highlighted by the investigation by Li and colleagues [228]. The study identified 29 dysregulated miRNAs within exosomes derived from two paclitaxel resistant prostate cancer cell line models [228].

## 4. Influences of lncRNAs in Cancer Chemoresistance

Notwithstanding the myriad of networking interactions leading to miRNA directed gene regulatory effects within cellular populations, the more recent discovery of a separate family of non-coding RNAs, namely lncRNAs, leads to the identification of an additional level of gene regulation within the human body. According to the latest version of LNCipedia, there are over 60,000 members of the lncRNA family that have been catalogued [234,235]. LncRNAs are non-coding RNA genes of at least 200 nucleotides long. LncRNAs can either act as positive or negative regulators of target gene expression, with this activity being directed either on transcripts originating from the same locus as the lncRNA itself (cis-acting) or directed on target transcripts originating on other loci (trans-acting) [6].

Table 2 and Supplementary Materials Table S2 highlight in detail the currently reported scientific evidence for the influence of multiple lncRNAs on varying cancer models. Interestingly, previous studies have highlighted the detrimental effects of one individual lncRNA, known as homeobox transcript antisense RNA (HOTAIR), having an elevated prevalence within multiple tumour chemoresistance phenotypes [236,237,238].

The investigation carried out by Fang and colleagues focused on the possible effects of HOTAIR on chemoresistance in small cell lung cancer, mainly through knock-down of the lncRNA in chemoresistant and parental cell line models, followed by viability assays [236]. Apart from confirming HOTAIR knock-down with enhanced chemosensitivity of the affected cell lines to doxorubicin, cisplatin and etoposide, the study also recognized the chemoresistance phenotype was additionally linked to increased methylation of homeobox A1 (HOXA1), suggesting HOTAIR influences in affecting such a methylation status [236]. The study also confirmed that HOTAIR inhibition, through short hairpin RNA antagonist employment in murine tumour xenograft models for small cell lung cancer, led to a reduction in tumour growth [236].

In a similar study conducted by Liu and colleagues, HOTAIR expression was discovered to be up-regulated in the cisplatin-resistant A549 lung adenocarcinoma cell line model, with consequent re-sensitisation of the cell line to cisplatin exposure following HOTAIR knock-down [237]. This short interfering RNA (siRNA)-induced HOTAIR knock-down effect was also linked to enhanced cell cycle arrest and apoptosis, together with a reduction in cell proliferation, through control of p21^WAF1/CIP1^ expression [237].

Furthermore, the study performed by Ozes and colleagues investigated the possible influences of HOTAIR on ovarian cancer chemoresistance properties, specifically for platinum-based chemotherapeutic agent chemoresistance [238]. The results of the study highlighted exacerbated HOTAIR expression within platinum drug resistant ovarian tumour samples when compared to primary ovarian tumour counterpart samples [238]. The study also revealed that HOTAIR up-regulation allows for prolonged NF-*K*B expression, leading to extended DNA damage response mechanisms to take place, following platinum-based drug exposure and therefore contributing to the chemoresistance phenotype development [238].

In addition to HOTAIR, another putative chemoresistance lncRNA of particular interest is MRUL (NR_024549), since the chromosomal locus for MRUL is in close proximity to the locus for the Multi Drug Resistance 1 (MDR1) gene—the latter being recognised as the most important gene to induce cancer chemoresistance phenotypes [239]. Such a study highlights the unique properties of lncRNAs in their capacity to perform cis-regulatory functions on neighbouring transcripts of clinical relevance.

Evidence for the regulatory role of lncRNAs in directing both cell proliferative signalling and drug modulation mechanisms affecting chemoresistance can be found in the study performed by Dong and colleagues [240]. This particular study recognised the effect of GAS5 in enhancing apoptosis due to gefitinib activity within innate EGFR tyrosine-kinase inhibitor (TKI) resistant lung adenocarcinomas (A549 cell line), through the gene regulating role of GAS5 on insulin-like growth factor 1 receptor (IGF-1R) [240]. Interestingly, the investigation conducted by Cheng and colleagues identified UCA1 as being upregulated in acquired (non T790M) EGFR-TKI resistant non-small cell lung cancer [241]. UCA1 knockdown assays confirmed that this lncRNA, when downregulated, allowed for increased gefitinib sensitivity and furthermore inhibited AKT/mTOR functions [241].

## 5. Conclusions and Perspectives

**Figure 1:**
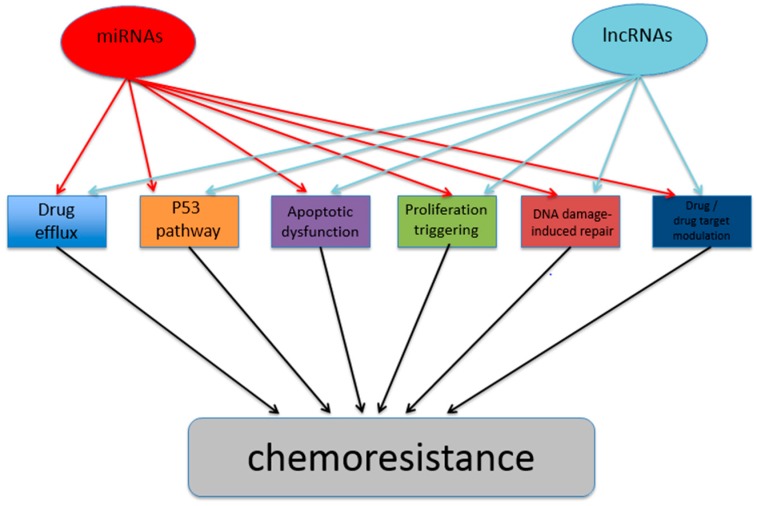
Model of miRNA and lncRNA influences on varying molecular pathway mechanisms leading to downstream effects on cancer chemoresistance phenotypes.

In essence, it can be stated that ncRNAs do have a place in regulating cancer chemoresistance properties, merely based on the body of evidence described above. Furthermore, the recent scientific literature on this niche research reveals that both miRNAs and lncRNAs have important roles in affecting the main mechanisms currently known to lead to the development of cancer chemoresistance phenotypes (see Figure 1).

Undoubtedly, the recent progress in molecular analytical and sequencing technologies has advanced to the levels that the entire miRnome/lncRNome can be quantified in a rapid and reliable manner, facilitating investigators’ efforts to identify unique expression profiles that are linked with defined tumour chemoresistance properties.

Such breakthroughs in technology are proving to be essential for biomarker researchers since evermore studies are leading to the paradigm that tumour clinical characteristics such as chemoresistance are the result of influence by multiple miRNAs and/or lncRNAs acting in a simultaneous manner, and not merely the outcome of one individual non-coding RNA’s dysregulated expression. The issue with this paradigm is the degree of complexity and resource consumption in carrying out detailed functional analyses to validate each permutation of non-coding RNA influences from an identified expression profile comprising just a handful of miRNAs/lncRNAs. Hopefully, further advances in bioinformatics and analytical technologies can permit more accurate trawling efforts to pinpoint such biomarkers and/or possibly allow for high throughput functional analyses for the entire miRnome/lncRNome in a rapid and effective manner.

The clinical importance for all global research efforts to identify and validate novel non-coding RNA biomarkers for cancer chemoresistance must certainly not be underestimated. The validation of reliable miRNA and/or lncRNA biomarkers for individual cancer chemoresistance (be it innate or acquired) can lead to the exploitation of such biomarkers as novel drug targets. Ultimately, antagonists and/or mimics (depending whether the miRNA/lncRNA is up- or down-regulated) for each non-coding RNA drug target can be developed and safely delivered as adjunct therapy together with conventional chemotherapeutic drugs. The adjuvant therapy leads to enhanced tumour sensitivity for the conventional chemotherapeutic drugs, therefore markedly enhancing chemotherapy effectiveness.

Alternatively, in patients who are particularly prone to the dose limiting adverse effects of conventional chemotherapy, the doses for the latter can be reduced due to the addition of the novel non-coding RNA-directed therapy. This leads to a great reduction in dose-limiting adverse effects and consequent discomfort in the cancer patient.

Finally, such chemoresistance biomarker expression profiles can be easily quantified from tumour biopsy through real time quantitative polymerase chain reaction (RT-qPCR) assays. The additional clinical information regarding the chemoresistance properties can provide the oncologist with valuable pre-emptive knowledge. Such additional information aids in developing a bespoke chemotherapy drug combination for the cancer patient that maximizes therapeutic efficacy and therefore minimises “trial and error” chemotherapy regimes, since the tumour would be exposed only to the chemotherapeutic agents to which it is fully sensitive.

However, efforts to render such a powerful theranostic technology is confronted with two main issues for it to become commonplace within global reach.

Firstly, the most effective technologies for accurate quantitative analysis of ncRNAs remain to be real-time, reverse transcription quantitative PCR (RT-qPCR) and next-generation sequencing. Both such technologies require sophisticated equipment and highly skilled staff dedicated to the processing of clinical samples for miRNA and lncRNA expression profiling. Eventually, these technologies can be miniaturized and simplified to the level of the development of a cost-effective point-of-care diagnostic apparatus that can be utilised by healthcare professionals with limited experience in the analytical technologies being employed.

Secondly, the issues regarding safe and effective drug delivery of novel miRNA and lncRNA therapeutics still pose a hurdle to rapid development of such translational medicine and effective availability for use by the individual cancer patient. Notwithstanding this issue however, the pharmaceutical industry are currently focusing hard on multiple circumvention methods for effectively providing efficient drug delivery options. These efforts are concentrated in pharmaceutical companies that are entirely dedicated to the research and development of miRNA and lncRNA-based therapeutics.

However, the authors sincerely believe that, despite such challenges, the advent of such novel clinical oncology drug treatment/management protocols will become a reality within the hospital setting in the not too distant future.

## Figures and Tables

**Table 1:** Compendium of miRNAs identified to influence cancer chemoresistance since 2013, either as oncomiRs or as tumour suppressors. A details list of additional such miRNAs, identified prior to 2013, can be obtained through the open-access review publication by Garofalo and Croce [42]. Furthermore, due to recent changes in miRNA annotation and nomenclature, the putative miRNAs mentioned in the literature have been listed according to the latest annotation changes on the miRBase repository, using the miRBase tracker webtool (annotation history of mature miRNA searches) [43]. Table keys: u, Upregulated; d, Downregulated; nd, not described; +, Increase; −, Reduction.

miRNA/s Involved (Species—*Homo sapiens*)	Accession (MIMAT) Number	Cancer Model	Affected Chemotherapeutic Drugs	Dysregulation Status	Effect on Chemo-Resistance Phenotype	Ref.
miR-34a-5p	0000255	bladder	neoadjuvant chemotherapy	nd	+	[44]
miR-100-5p	0000098					
miR-146b-5p	0002809					
miR-9-5p	0000441					
miR-193a-3p	0000459					
let-7c-5p	0000064	bladder	Platinum-based neoadjuvant chemotherapy	d	+	[45]
miR-1290	0005880	bladder	gemcitabine	u	+	[46]
miR-138-5p	0000430			u	+	
let-7i-5p	0000415			d	+	
let-7b-5p	0000063			d	+	
miR-193a-3p	0000459	bladder	MDR	u	+	[47,48,49]
miR-21-5p	0000076	breast	gemcitabine	u	+	[50]
miR-25-3p	0000081	breast		d	−	[51]
miR-125b-5p	0000423	breast		u	+	[52]
miR-149-5p	0000450	breast		d	+	[53]
miR-320a	0000510	breast		d	+	[54]
miR-29a-3p	0000086	breast	doxorubicin	u	+	[55]
miR-129-2-3p	0004605	breast	docetaxel	u	+	[56]
miR-139-5p	0000250	breast	docetaxel	d	+	[57]
miR-760	0004957	breast	doxorubicin	u	+	[58]
miR-484	0002174	breast		u	+	[59]
miR-223-3p	0000280	breast		d	+	[60]
miR-489-3p	0002805	breast		u	-	[61]
miR-34a-5p	0000255	breast	doxorubicin docetaxel	u	−	[62]
miR-222-3p	0000279			d	−	
miR-452-5p	0001635			d	−	
miR-29a-3p	0000086			d	−	
let-7a-5p	0000062	breast	epirubicin	d	+	[63]
miR-181b-5p	0000257	breast	doxorubicin	u	+	[64]
miR-141-3p	0000432	breast	docetaxel	u	+	[65]
miR-145-5p	0000437	breast	doxorubicin	u	−	[66]
miR-100-5p	0000098	breast	doxorubicin docetaxel	u	+	[67]
miR-222-3p	0000279			u	+	
miR-30a-3p	0000088			u	+	
miR-30a-5p	0000087			u	+	
miR-30c-5p	0000244	breast		u	−	[68]
miR-155-5p	0000646	breast	tamoxifen	u	+	[69]
miR-663a	0003326	breast	doxorubicin	u	+	[70]
miR-302a-3p	0000684	breast	doxorubicin	u	−	[71]
miR-302b-3p	0000715			u	−	
miR-302c-3p	0000717			u	−	
miR-302d-3p	0000718			u	−	
miR-200c-3p	0000617	breast	doxorubicin	u	−	[72]
miR-181a-5p	0000256	cervical	cisplatin	u	+	[73]
miR-125a-5p	0000443	cervical	paclitaxel	u	−	[74]
miR-100-5p	0000098	chondrosarcoma	cisplatin	u	−	[75]
miR-4299	0016851	colon	capecitabine oxaliplatin	d	−	[76]
miR-196b-5p	0001080			u	−	
miR-34a-5p	0000255	colon	5-fluorouracil	u	−	[77]
miR-122-5p	0000421	colon	5-fluorouracil	u	−	[78]
miR-409-3p	0001639	colon	oxaliplatin	u	−	[79]
miR-223-3p	0000280	colon		d	+	[60]
miR-494-3p	0002816	colon	5-fluorouracil	u	−	[80]
miR-125a-5p	0000443	colon	paclitaxel	u	−	[81]
miR-125b-5p	0000423					
miR-218-5p	0000275	colorectal	5-fluorouracil	u	−	[82]
miR-203a-3p	0000264	colorectal	paclitaxel 5-fluorouracil	u	−	[83,84]
miR-1914-3p	0007890	colorectal	capecitabine oxaliplatin	u	−	[85]
miR-1915-3p	0007892			u	−	
miR-204-5p	0000265	colorectal	5-fluorouracil	u	−	[86]
miR-139-5p	0000250	colorectal	5-fluorouracil	u	−	[87]
miR-205-5p	0000266	colorectal		u	+	[88]
miR-373-3p	0000726			u	+	
miR-425-5p	0003393	colorectal	5-fluorouracil oxaliplatin	u	+	[89]
miR-429	0001536	colorectal	5-fluorouracil	u	+	[90]
miR-34a-5p	0000255	colorectal	5-fluorouracil	u	−	[91]
miR-519c-3p	0002832	colorectal	5-fluorouracil irinotecan	d	+	[92]
miR-520g-3p	0002858	colorectal	5-fluorouracil	u	+	[93]
miR-23a-3p	0000078	colorectal	5-fluorouracil	u	+	[94]
miR-96-5p	0000095	colorectal	5-fluorouracil	u	−	[95]
miR-587	0003253	colorectal	5-fluorouracil	u	+	[96]
miR-218-5p	0000275	endometrial	paclitaxel	u	−	[97]
miR-125b-5p	0000423	ewing sarcoma	doxorubicin	u	+	[98]
miR-145-5p	0000437	gallbladder	cisplatin	u	−	[99]
miR-1284	0005941	gastric	vincristine	u	−	[100]
miR-375	0000728	gastric	cisplatin	u	−	[101]
miR-23b-3p	0000418	gastric	MDR	u	−	[102]
miR-20a-5p	0000075	gastric	cisplatin	u	+	[103]
miR-34c-5p	0000686	gastric	paclitaxel	d	+	[104]
miR-16-5p	0000069	gastric	etoposide 5-fluorouracil	u	−	[105]
miR-9-5p	0000441	glioblastoma	temozolomide	u	+	[106]
miR-20a-5p	0000075	glioblastoma	temozolomide	u	−	[107]
miR-21-5p	0000076	glioblastoma	doxorubicin	u	+	[108]
miR-873-5p	0004953	glioblastoma	cisplatin	u	−	[109]
miR-210-3p	0000267	glioblastoma	temozolomide	u	−	[110]
miR-138-5p	0000430	glioblastoma	temozolomide	u	+	[111]
miR-125b-5p	0000423	glioblastoma	temozolomide	u	−	[112]
miR-203a-3p	0000264	glioblastoma		d	+	[113]
let-7b-5p	0000063	glioblastoma	cisplatin	d	+	[114]
miR-181b-5p	0000257	glioma	temozolomide	u	−	[115]
miR-124-3p	0000422	glioma	temozolomide	u	−	[116]
miR-200a-3p	0000682	glioma	temozolomide	u	−	[117]
miR-136-5p	0000448	glioma	cisplatin	u	−	[118]
miR-10b-5p	0000254	head/neck squamous cell	cisplatin	u	+	[119]
miR-21-5p	0000076	hepatocellular		u	+	[120]
miR-34a-5p	0000255	hepatocellular	sorafenib	u	−	[121]
miR-26b-5p	0000083	hepatocellular	doxorubicin	u	−	[122]
miR-106a-5p	0000103	hepatocellular	gemcitabine	d	+	[123]
miR-101-3p	0000099	hepatocellular	cisplatin	u	−	[124]
miR-125b-5p	0000423	hepatocellular	5-fluorouracil	u	−	[125]
miR-145-5p	0000437	hepatocellular	doxorubicin	u	−	[126]
miR-141-3p	0000432	hepatocellular	5-fluorouracil	u	+	[127]
miR-122-5p	0000421	hepatocellular	sorafenib	d	+	[128]
miR-340-5p	0004692	hepatocellular	cisplatin	u	−	[129]
miR-182-5p	0000259	hepatocellular	cisplatin	u	+	[130]
miR-215-5p	0000272	hepatocellular	doxorubicin	u	+	[131]
miR-135b-5p	0000758	leukaemia	genotoxic agent treatment (eg., etoposide, doxorubicin)	u	+	[132]
miR-196b-5p	0001080			u	+	
miR-17-3p	0000071	leukaemia		d	−	[133]
miR-17-5p	0000070			d	−	
miR-20a-5p	0000075			d	−	
miR-21-5p	0000076	leukaemia	etoposide, doxorubicin	d	−	[134]
miR-181a-5p	0000256	leukaemia	doxorubicin	u	+	[135]
miR-181c-5p	0000258	leukaemia	chronic myelocytic leukaemia	u	−	[136]
let-7a-5p	0000062	leukaemia	cytarabine	d	+	[137]
let-7c-5p	0000064	lung	cisplatin	u	−	[138]
miR-1244	0005896	lung	cisplatin	u	−	[139]
miR-96-5p	0000095	lung	cisplatin	u	+	[140]
miR-107	0000104	lung	cisplatin	u	−	[141]
miR-378a-3p	0000732	lung	cisplatin	u	−	[142]
miR-192-5p	0000222	lung	cisplatin	u	+	[143]
miR-205-5p	0000266	lung		u	+	[144]
miR-21-5p	0000076	lung	cisplatin	d	−	[145]
miR-24-3p	0000080	lung	etoposide, cisplatin	d	+	[146]
miR-299-3p	0000687	lung	doxorubicin	u	−	[147]
miR-27a-3p	0000084	lung	cisplatin	u	−	[148]
miR-551a	0003214	lung		u	+	[149]
miR-100-5p	0000098	lung		u	+	[150]
miR-146a-5p	0000449	lung	cisplatin	u	+	[151]
miR-182	(sequence not listed in paper)	lung	cisplatin	u	+	[152]
miR-650	0003320	lung	docetaxel	u	+	[153]
miR-224-5p	0000281	lung	cisplatin	u	+	[154]
miR-451a	0001631	lung	docetaxel	u	−	[155]
miR-15b-5p	0000417	lung	cisplatin	u	−	[156]
miR-148b-3p	0000759	lung	cisplatin	u	−	[157]
miR-205-5p	0000266	lung	carboplatin	u	+	[158]
miR-218-5p	0000275			u	+	
miR-26b-5p	0000083	lung		d	−	[159]
miR-192-5p	0000222	lung	gemcitabine, cisplatin	u	−	[160]
miR-197-3p	0000227	lung	platinum-based	d	+	[161]
miR-7-5p	0000252	lung		u	−	[162]
miR-940	0004983	lung	cisplatin	d	+	[163]
miR-200b-3p	0000318	lung	docetaxel	u	−	[164]
miR-200c-3p	0000617	lung	methotrexate	u	−	[165]
miR-494-3p	0002816	lung		u	−	[166]
miR-377-3p	0000730	lymphoma (b-cell)	venetoclax	u	+	[167]
miR-125b-5p	0000423	lymphoma (b-cell)	cyclophosphamide, doxorubicin, vincristine	u	+	[168]
miR-130a-3p	0000425			u	+	
miR-21-5p	0000076	nasopharyngeal	cisplatin	u	+	[169]
miR-634	0003304	nasopharyngeal	paclitaxel	u	−	[170]
miR-214-3p	0000271	oesophageal (squamous cell)	cisplatin	u	−	[171]
miR-21-5p	0000076	oesophageal (squamous cell)	5-fluorouracil cisplatin (circulating miRnas)	u	+	[172]
miR-193a-3p	0000459	oesophageal	chemoradiation	u	−	[173]
miR-27a-3p	0000084	oesophageal	cisplatin	u	+	[174]
miR-221-3p	0000278	oesophageal	5-fluorouracil	u	+	[175]
miR-181a-5p	0000256	oral squamous cell	cisplatin	u	−	[176]
miR-23a-3p	0000078	oral squamous cell	cisplatin	u	+	[177]
miR-143-3p	0000435	osteosarcoma	doxorubicin	d	+	[178]
miR-101-3p	0000099	osteosarcoma	cisplatin doxorubicin methotrexate	u	−	[179]
miR-29b-1	MI00000105 (precursor)	osteosarcoma		u	−	[180]
miR-33a-5p	0000091	osteosarcoma	cisplatin	u	+	[181]
miR-34c-5p	0000686	osteosarcoma		u	−	[182]
miR-301a-3p	0000688	osteosarcoma	doxorubicin	u	+	[183]
miR-22-3p	0000077	osteosarcoma		u	−	[184]
miR-382-5p	0000737	osteosarcoma		u	−	[185]
miR-193a-5p	0004614	osteo-/ewing sarcoma	cisplatin	u	−	[186]
miR-136-5p	0000448	ovarian	cisplatin	u	+	[187]
miR-30a-5p	0000087	ovarian	cisplatin	u	−	[188]
miR-149-5p	0000450	ovarian	paclitaxel	d	+	[189]
miR-9-5p	0000441	ovarian	paclitaxel	d	+	[190]
miR-21-3p	0004494	ovarian	cisplatin	u	+	[191]
miR-31-5p	0000089	ovarian	cisplatin	u	+	[192]
miR-31-5p	0000089	ovarian	taxane	u	−	[193]
miR-29b-3p	0000100	ovarian	paclitaxel	d	+	[194]
miR-200a-3p	0000682	ovarian	paclitaxel	u	−	[195]
miR-506-3p	0002878	ovarian	cisplatin olaparib	u	−	[196]
miR-433-3p	0001627	ovarian	paclitaxel	u	+	[197]
miR-186-5p	0000456	ovarian	cisplatin	u	−	[198]
miR-1307-3p	0005951	ovarian		u	+	[199]
miR-224-5p	0000281	ovarian	cisplatin	u	+	[200]
miR-130a-3p	0000425	ovarian	cisplatin	u	−	[201]
miR-374a-5p	0000727			u	−	
miR-106a-5p	0000103	ovarian	cisplatin	u	−	[202]
miR-106a-5p	0000103	ovarian	paclitaxel	u	+	[203]
miR-591	0003259			d	+	
miR-770-5p	0003948	ovarian	cisplatin	u	−	[204]
miR-21-5p	0000076	ovarian	paclitaxel; exosome-driven	u	+	[205]
miR-199b-5p	0000263	ovarian	cisplatin	d	+	[206]
miR-145-5p	0000437	ovarian	paclitaxel	u	−	[207]
let-7e-5p	0000066	ovarian	cisplatin	d	+	[208]
miR-152-3p	0000438	ovarian	cisplatin	u	−	[209]
miR-128-3p	0000424	ovarian	cisplatin	d	+	[210]
miR-484	0002174	ovarian		d	+	[211]
miR-642a-5p	0003312			d	+	
miR-217	0000274			d	+	
miR-23a-3p	0000078	ovarian		u	+	[212]
miR-27b-3p	0000419			u	+	
miR-424-5p	0001341			u	+	
miR-503-5p	0002874			u	+	
miR-21-5p	0000076	ovarian	carboplatin paclitaxel	u	+	[213]
miR-214-3p	0000271			u	+	
miR-182-5p	0000259	ovarian	cisplatin paclitaxel	u	+	[214]
miR-200c-3p	0000617	pancreatic		u	-	[215]
miR-33a-5p	0000091	pancreatic	gemcitabine	u	-	[216]
miR-17-92 cluster		pancreatic		d	+	[217]
miR-221-3p	0000278	pancreatic	5-fluorouracil	u	+	[218]
miR-1246	0005898	pancreatic		u	+	[219]
miR-181b-5p	0000257	pancreatic	gemcitabine	u	+	[220]
miR-494-3p	0002816	pancreatic		u	−	[221]
miR-101-3p	0000099	pancreatic	gemcitabine	u	−	[222]
miR-100-5p	0000098	pancreatic (ductal)	gemcitabine	u	+	[223,224]
miR-21-5p	0000076			u	+	
miR-99a-5p	0000097			u	+	
miR-125b-5p	0000423			u	+	
miR-138-5p	0000430			u	+	
miR-210-3p	0000267			u	+	
miR-31-3p	0004504			d	+	
miR-330-3p	0000751			d	+	
miR-378-5p	0000731			d	+	
let-7a-5p	0000062	pancreatic	gemcitabine	u	−	[225]
miR-205-5p	0000266	pancreatic	gemcitabine	u	−	[226]
miR-506-3p	0002878	pancreatic		d	+	[227]
miR-3176	0015053	prostate	paclitaxel	d	+	[228]
miR-141-3p	0000432			d	+	
miR-5004-5p	0021027			d	+	
miR-16-5p	0000069			d	+	
miR-3915	0018189			d	+	
miR-488-3p	0004763			d	+	
miR-23c	0018000			d	+	
miR-3673	0018096			d	+	
miR-3654	0018074			d	+	
miR-32-5p	0000090			u	+	
miR-606	0003274			u	+	
miR-381-3p	0000736			u	+	
miR-429	0001536			u	+	
miR-708	0004926	renal	doxorubicin	u	−	[229]
let-7b-5p	0000063	renal	5-fluorouracil	u	−	[230]
let-7c-5p	0000064			u	−	
miR-200c-3p	0000617	renal	docetaxel	d	+	[231]

**Table 2:** Compendium of lncRNAs identified to influence cancer chemoresistance, either as oncogenic lncRNAs or as tumour suppressors. Table keys: u, Upregulated; d, Downregulated; +, Exacerbation; −, Inhibition.

lncRNA/s involved (Species—*Homo sapiens*)	gene ID (LNCipedia.org—Where Applicable)	Cancer Model	Affected Chemo-Therapy Drugs	Dysregulation Status	Effect on Chemo-Resistance Phenotype	Ref.
UCA1	UCA1	bladder	cisplatin, gemcitabine	u	+	[242,243]
NONHSAT028712	lnc-DGKA-1	breast	doxorubicin	u	+	[244]
NONHSAT057282	lnc-RP11-677O4.1.1-7			u	+	
NONHSAG023333	lnc-TXNDC2-7			u	+	
ARA	lnc-ALG13-7	breast	doxorubicin	u	+	[245]
ATB	lncRNA-AL589182	breast	trastuzumab	u	+	[246]
GAS5	GAS5	breast	trastuzumab	d	+	[247]
XIST	XIST	breast	alkylating agents	u	+	[248]
53BP1	Lnc-TP53BP1-1			d	+	
CCAT2	lnc-POU5F1B-8	breast	5-fluorouracil	u	+	[249]
snaR	lnc-BSPH1-1/2	colon	5-fluorouracil	u	+	[250]
LINC00152	LINC00152	colon	oxaliplatin	u	+	[251]
SLC25A25-AS1	SLC25A25-AS1	colorectal		d	+	[252]
MRUL	(NR_024549)	gastric	MDR	u	+	[239]
AK022798	lnc-TRAF3IP3-3	gastric	cisplatin	u	+	[253]
PVT1	PVT1	gastric	MDR	u	+	[254]
LINC-ROR	LINC-ROR	hepatocellular	sorafenib, doxorubicin	u	+	[255]
CCAT1	lnc-TMEM75-3	lung	docetaxel	u	+	[256]
AK126698	(LINC00969)	lung	cisplatin	d	+	[257]
HOTAIR	HOTAIR	lung	MDR	u	+	[236,237]
GAS5	GAS5	lung	EGFR-tyrosine kinase inhibitors	u	−	[240]
UCA1	UCA1	lung	EGFR-tyrosine kinase inhibitors	u	+	[241]
MEG3	MEG3	lung	cisplatin	u	+	[258]
GAS5	GAS5	lymphoma (mantle cell)	mTOR inhibitors	u	−	[259]
N375709	(lnc-SRCIN1-1)	nasopharyngeal	paclitaxel	d	−	[260]
LINC-ROR	LINC-ROR	nasopharyngeal		u	+	[261]
TUG1	TUG1	oesophageal		u	+	[262]
LINC00161	LINC00161	osteosarcoma	cisplatin	u	−	[263]
ODRUL	FOXC2-AS1	osteosarcoma	doxorubicin	u	+	[264]
ODRUL	FOXC2-AS1	osteosarcoma	doxorubicin	u	+	[265]
HOTAIR	HOTAIR	ovarian	platinum-based drugs	u	+	[238]
PVT1	PVT1	ovarian	cisplatin	u	+	[266]
UCA1	UCA1	ovarian		u	+	[267]
HOTTIP	BCYRN1	ovarian	carboplatin	d	+	[268]
HOTTIP	BCYRN1	pancreatic	gemcitabine	u	+	[269]
PVT1	PVT1	pancreatic	gemcitabine	u	+	[270]
MALAT-1	MALAT1	pancreatic		u	+	[271]
GAS5	GAS5	prostate	mTOR inhibitors	u	−	[272]
UCA1	UCA1	breast	tamoxifen	u	+	[273]

## Supplementary Materials

The following are available online at www.mdpi.com/2073-4425/8/3/95/s1, Table S1: Frequency chart depicting prevalence of individual miRNAs in specific cancer models reported in the literature (as depicted in Table 1 above) and from the data reported in the review article by Garofalo and Croce [42], Table S2: Frequency chart depicting prevalence of individual lncRNAs in specific cancer models reported in the literature (as depicted in Table 2 above).

## References

[1] Ayers D., Day P.J. (2009). Unlocking the potential of RNA interference as a therapeutic tool. Malta Med. J..

[2] Ambros V. (2001). microRNAs: Tiny regulators with great potential. Cell.

[3] Ayers D., Baron B., Hunter T. (2015). miRNA Influences in NRF2 Pathway Interactions within cancer models. J. Nucleic Acids.

[4] Stallings R.L. (2009). MicroRNA involvement in the pathogenesis of neuroblastoma: Potential for microRNA mediated therapeutics. Curr. Pharm. Des..

[5] Ayers D. (2011). Implications of miRNA-directed gene silencing in cancer. RNAi Technology.

[6] Ayers D. (2013). Long Non-Coding RNAs: Novel Emergent Biomarkers for Cancer Diagnostics. J. Cancer Res. Treat..

[7] Ayers D., Nasti A. (2012). Utilisation of nanoparticle technology in cancer chemoresistance. J. Drug Deliv..

[8] Lage H. (2016). Gene Therapeutic Approaches to Overcome ABCB1-Mediated Drug Resistance. Recent Results Cancer Res..

[9] Schmitt S.M., Stefan K., Wiese M. (2017). Pyrrolopyrimidine derivatives and purine analogs as novel activators of Multidrug Resistance-associated Protein 1 (MRP1, ABCC1). Biochim. Biophys. Acta.

[10] Robey R.W., Massey P.R., Amiri-Kordestani L., Bates S.E. (2010). ABC transporters: Unvalidated therapeutic targets in cancer and the CNS. Anticancer Agents Med. Chem..

[11] Krishna R., Mayer L.D. (2000). Multidrug resistance (MDR) in cancer. Mechanisms, reversal using modulators of MDR and the role of MDR modulators in influencing the pharmacokinetics of anticancer drugs. Eur. J. Pharm. Sci..

[12] Colone M., Calcabrini A., Toccacieli L., Bozzuto G., Stringaro A., Gentile M., Cianfriglia M., Ciervo A., Caraglia M., Budillon A. (2008). The multidrug transporter P-glycoprotein: A mediator of melanoma invasion?. J. Investig. Dermatol..

[13] MacLaine N.J., Hupp T.R. (2011). How phosphorylation controls p53. Cell Cycle.

[14] Macchiarulo A., Giacchè N., Mancini F., Puxeddu E., Moretti F., Pellicciari R. (2011). Alternative strategies for targeting mouse double minute 2 activity with small molecules: Novel patents on the horizon?. Expert Opin. Ther. Pat..

[15] Mogi A., Kuwano H. (2011). TP53 mutations in nonsmall cell lung cancer. J. Biomed. Biotechnol..

[16] Plati J., Bucur O., Khosravi-Far R. (2011). Apoptotic cell signaling in cancer progression and therapy. Integr. Biol. Quant. Biosci. Nano Macro.

[17] Rolland S.G., Conradt B. (2010). New role of the BCL2 family of proteins in the regulation of mitochondrial dynamics. Curr. Opin. Cell Biol..

[18] Gandhi L., Camidge D.R., Ribeiro de Oliveira M., Bonomi P., Gandara D., Khaira D., Hann C.L., McKeegan E.M., Litvinovich E., Hemken P.M. (2011). Phase I study of Navitoclax (ABT-263), a novel Bcl-2 family inhibitor, in patients with small-cell lung cancer and other solid tumors. J. Clin. Oncol..

[19] Yu Z., Wang R., Xu L., Xie S., Dong J., Jing Y. (2011). β-Elemene piperazine derivatives induce apoptosis in human leukemia cells through downregulation of c-FLIP and generation of ROS. PLoS ONE.

[20] Avraham R., Yarden Y. (2011). Feedback regulation of EGFR signalling: Decision making by early and delayed loops. Nat. Rev. Mol. Cell Biol..

[21] Chuang S.E., Yeh P.Y., Lu Y.S., Lai G.M., Liao C.M., Gao M., Cheng A.L. (2002). Basal levels and patterns of anticancer drug-induced activation of nuclear factor-kappaB (NF-κB), and its attenuation by tamoxifen, dexamethasone, and curcumin in carcinoma cells. Biochem. Pharmacol..

[22] Olmos Y., Brosens J.J., Lam E.W.-F. (2011). Interplay between SIRT proteins and tumour suppressor transcription factors in chemotherapeutic resistance of cancer. Drug Resist. Updat..

[23] Arora S., Kothandapani A., Tillison K., Kalman-Maltese V., Patrick S.M. (2010). Downregulation of XPF-ERCC1 enhances cisplatin efficacy in cancer cells. DNA Repair.

[24] Kim H., An J.Y., Noh S.H., Shin S.K., Lee Y.C., Kim H. (2011). High microsatellite instability predicts good prognosis in intestinal-type gastric cancers. J. Gastroenterol. Hepatol..

[25] Martin L.P., Hamilton T.C., Schilder R.J. (2008). Platinum resistance: The role of DNA repair pathways. Clin. Cancer Res..

[26] Assaraf Y.G. (2007). Molecular basis of antifolate resistance. Cancer Metastasis Rev..

[27] Yeung J., Esposito M.T., Gandillet A., Zeisig B.B., Griessinger E., Bonnet D., So C.W.E. (2010). β-Catenin mediates the establishment and drug resistance of MLL leukemic stem cells. Cancer Cell.

[28] Ren J., Singh B.N., Huang Q., Li Z., Gao Y., Mishra P., Hwa Y.L., Li J., Dowdy S.C., Jiang S.-W. (2011). DNA hypermethylation as a chemotherapy target. Cell. Signal..

[29] Banerjee Mustafi S., Chakraborty P.K., Naz S., Dwivedi S.K.D., Street M., Basak R., Yang D., Ding K., Mukherjee P., Bhattacharya R. (2016). MDR1 mediated chemoresistance: BMI1 and TIP60 in action. Biochim. Biophys. Acta.

[30] Lee M.-R., Ji S.-Y., Mia-Jan K., Cho M.-Y. (2015). Chemoresistance of CD133(+) colon cancer may be related with increased survivin expression. Biochem. Biophys. Res. Commun..

[31] Xia L.-L., Tang Y.-B., Song F.-F., Xu L., Ji P., Wang S.-J., Zhu J.-M., Zhang Y., Zhao G.-P., Wang Y. (2016). DCTPP1 attenuates the sensitivity of human gastric cancer cells to 5-fluorouracil by up-regulating MDR1 expression epigenetically. Oncotarget.

[32] Yao J., Wei X., Lu Y. (2016). Chaetominine reduces MRP1-mediated drug resistance via inhibiting PI3K/Akt/Nrf2 signaling pathway in K562/Adr human leukemia cells. Biochem. Biophys. Res. Commun..

[33] Zhan M., Wang H., Chen T., Chen W., Yang L., He M., Xu S., Wang J. (2015). NOX1 mediates chemoresistance via HIF1α/MDR1 pathway in gallbladder cancer. Biochem. Biophys. Res. Commun..

[34] Gibb E.A., Brown C.J., Lam W.L. (2011). The functional role of long non-coding RNA in human carcinomas. Mol. Cancer.

[35] Gibb E.A., Enfield K.S.S., Stewart G.L., Lonergan K.M., Chari R., Ng R.T., Zhang L., MacAulay C.E., Rosin M.P., Lam W.L. (2011). Long non-coding RNAs are expressed in oral mucosa and altered in oral premalignant lesions. Oral Oncol..

[36] Lai E.C. (2002). Micro RNAs are complementary to 3’ UTR sequence motifs that mediate negative post-transcriptional regulation. Nat. Genet..

[37] McManus M.T., Sharp P.A. (2002). Gene silencing in mammals by small interfering RNAs. Nat. Rev. Genet..

[38] Ponting C.P., Oliver P.L., Reik W. (2009). Evolution and functions of long noncoding RNAs. Cell.

[39] Shamovsky I., Nudler E. (2006). Gene control by large noncoding RNAs. Sci. STKE.

[40] Carta A., Chetcuti R., Ayers D. (2014). An Introspective Update on the Influence of miRNAs in Breast Carcinoma and Neuroblastoma Chemoresistance. Genet. Res. Int..

[41] Griffiths-Jones S., Grocock R.J., van Dongen S., Bateman A., Enright A.J. (2006). miRBase: MicroRNA sequences, targets and gene nomenclature. Nucleic Acids Res..

[42] Garofalo M., Croce C.M. (2013). MicroRNAs as therapeutic targets in chemoresistance. Rev. Comment. Antimicrob. Anticancer Chemother..

[43] Van Peer G., Lefever S., Anckaert J., Beckers A., Rihani A., Van Goethem A., Volders P.-J., Zeka F., Ongenaert M., Mestdagh P. (2014). miRBase Tracker: Keeping track of microRNA annotation changes. Database J. Biol. Databases Curation.

[44] Amir S., Mabjeesh N.J. (2017). microRNA expression profiles as decision-making biomarkers in the management of bladder cancer. Histol. Histopathol..

[45] Vinall R.L., Tepper C.G., Ripoll A.A.Z., Gandour-Edwards R.F., Durbin-Johnson B.P., Yap S.A., Ghosh P.M., deVere White R.W. (2016). Decreased expression of let-7c is associated with non-response of muscle-invasive bladder cancer patients to neoadjuvant chemotherapy. Genes Cancer.

[46] Kozinn S.I., Harty N.J., Delong J.M., Deliyiannis C., Logvinenko T., Summerhayes I.C., Libertino J.A., Holway A.H., Rieger-Christ K.M. (2013). MicroRNA Profile to Predict Gemcitabine Resistance in Bladder Carcinoma Cell Lines. Genes Cancer.

[47] Deng H., Lv L., Li Y., Zhang C., Meng F., Pu Y., Xiao J., Qian L., Zhao W., Liu Q. (2015). The miR-193a-3p regulated PSEN1 gene suppresses the multi-chemoresistance of bladder cancer. Biochim. Biophys. Acta.

[48] Deng H., Lv L., Li Y., Zhang C., Meng F., Pu Y., Xiao J., Qian L., Zhao W., Liu Q. (2014). miR-193a-3p regulates the multi-drug resistance of bladder cancer by targeting the LOXL4 gene and the oxidative stress pathway. Mol. Cancer.

[49] Li Y., Deng H., Lv L., Zhang C., Qian L., Xiao J., Zhao W., Liu Q., Zhang D., Wang Y. (2015). The miR-193a-3p-regulated ING5 gene activates the DNA damage response pathway and inhibits multi-chemoresistance in bladder cancer. Oncotarget.

[50] Wu Z.-H., Tao Z.-H., Zhang J., Li T., Ni C., Xie J., Zhang J.-F., Hu X.-C. (2016). MiRNA-21 induces epithelial to mesenchymal transition and gemcitabine resistance via the PTEN/AKT pathway in breast cancer. Tumour Biol..

[51] Wang Z., Wang N., Liu P., Chen Q., Situ H., Xie T., Zhang J., Peng C., Lin Y., Chen J. (2014). MicroRNA-25 regulates chemoresistance-associated autophagy in breast cancer cells, a process modulated by the natural autophagy inducer isoliquiritigenin. Oncotarget.

[52] Wang H.-J., Guo Y.-Q., Tan G., Dong L., Cheng L., Li K.-J., Wang Z.-Y., Luo H.-F. (2013). miR-125b regulates side population in breast cancer and confers a chemoresistant phenotype. J. Cell. Biochem..

[53] He D.-X., Gu X.-T., Li Y.-R., Jiang L., Jin J., Ma X. (2014). Methylation-regulated miR-149 modulates chemoresistance by targeting GlcNAc N-deacetylase/N-sulfotransferase-1 in human breast cancer. FEBS J..

[54] He D.-X., Gu X.-T., Jiang L., Jin J., Ma X. (2014). A methylation-based regulatory network for microRNA 320a in chemoresistant breast cancer. Mol. Pharmacol..

[55] Shen H., Li L., Yang S., Wang D., Zhong S., Zhao J., Tang J. (2016). MicroRNA-29a contributes to drug-resistance of breast cancer cells to adriamycin through PTEN/AKT/GSK3β signaling pathway. Gene.

[56] Zhang Y., Wang Y., Wei Y., Li M., Yu S., Ye M., Zhang H., Chen S., Liu W., Zhang J. (2015). MiR-129-3p promotes docetaxel resistance of breast cancer cells via CP110 inhibition. Sci. Rep..

[57] Zhang H., Sun D.-W., Mao L., Zhang J., Jiang L.-H., Li J., Wu Y., Ji H., Chen W., Wang J. (2015). MiR-139-5p inhibits the biological function of breast cancer cells by targeting Notch1 and mediates chemosensitivity to docetaxel. Biochem. Biophys. Res. Commun..

[58] Lv J., Fu Z., Shi M., Xia K., Ji C., Xu P., Lv M., Pan B., Dai L., Xie H. (2015). Systematic analysis of gene expression pattern in has-miR-760 overexpressed resistance of the MCF-7 human breast cancer cell to doxorubicin. Biomed. Pharmacother. Bioméd. Pharmacothér..

[59] Ye F.-G., Song C.-G., Cao Z.-G., Xia C., Chen D.-N., Chen L., Li S., Qiao F., Ling H., Yao L. (2015). Cytidine Deaminase Axis Modulated by miR-484 Differentially Regulates Cell Proliferation and Chemoresistance in Breast Cancer. Cancer Res..

[60] Masciarelli S., Fontemaggi G., Di Agostino S., Donzelli S., Carcarino E., Strano S., Blandino G. (2014). Gain-of-function mutant p53 downregulates miR-223 contributing to chemoresistance of cultured tumor cells. Oncogene.

[61] Chen X., Wang Y.-W., Xing A.-Y., Xiang S., Shi D.-B., Liu L., Li Y.-X., Gao P. (2016). Suppression of SPIN1-mediated PI3K-Akt pathway by miR-489 increases chemosensitivity in breast cancer. J. Pathol..

[62] Zhang J., Zhang H., Chen L., Sun D.W., Mao C., Chen W., Wu J.Z., Zhong S.L., Zhao J.H., Tang J.H. (2014). β-elemene reverses chemoresistance of breast cancer via regulating MDR-related microRNA expression. Cell. Physiol. Biochem..

[63] Wu J., Li S., Jia W., Deng H., Chen K., Zhu L., Yu F., Su F. (2015). Reduced Let-7a Is Associated with Chemoresistance in Primary Breast Cancer. PLoS ONE.

[64] Zheng Y., Lv X., Wang X., Wang B., Shao X., Huang Y., Shi L., Chen Z., Huang J., Huang P. (2016). MiR-181b promotes chemoresistance in breast cancer by regulating Bim expression. Oncol. Rep..

[65] Yao Y.-S., Qiu W.-S., Yao R.-Y., Zhang Q., Zhuang L.-K., Zhou F., Sun L.-B., Yue L. (2015). miR-141 confers docetaxel chemoresistance of breast cancer cells via regulation of EIF4E expression. Oncol. Rep..

[66] Gao M., Miao L., Liu M., Li C., Yu C., Yan H., Yin Y., Wang Y., Qi X., Ren J. (2016). miR-145 sensitizes breast cancer to doxorubicin by targeting multidrug resistance-associated protein-1. Oncotarget.

[67] Chen W., Liu X., Lv M., Chen L., Zhao J., Zhong S., Ji M., Hu Q., Luo Z., Wu J. (2014). Exosomes from drug-resistant breast cancer cells transmit chemoresistance by a horizontal transfer of microRNAs. PLoS ONE.

[68] Bockhorn J., Dalton R., Nwachukwu C., Huang S., Prat A., Yee K., Chang Y.-F., Huo D., Wen Y., Swanson K.E. (2013). MicroRNA-30c inhibits human breast tumour chemotherapy resistance by regulating TWF1 and IL-11. Nat. Commun..

[69] Shen R., Wang Y., Wang C.-X., Yin M., Liu H.-L., Chen J.-P., Han J.-Q., Wang W.-B. (2015). MiRNA-155 mediates TAM resistance by modulating SOCS6-STAT3 signalling pathway in breast cancer. Am. J. Transl. Res..

[70] Hu H., Li S., Cui X., Lv X., Jiao Y., Yu F., Yao H., Song E., Chen Y., Wang M., Lin L. (2013). The overexpression of hypomethylated miR-663 induces chemotherapy resistance in human breast cancer cells by targeting heparin sulfate proteoglycan 2 (HSPG2). J. Biol. Chem..

[71] Zhao L., Wang Y., Jiang L., He M., Bai X., Yu L., Wei M. (2016). MiR-302a/b/c/d cooperatively sensitizes breast cancer cells to adriamycin via suppressing P-glycoprotein(P-gp) by targeting MAP/ERK kinase kinase 1 (MEKK1). J. Exp. Clin. Cancer Res..

[72] Kopp F., Oak P.S., Wagner E., Roidl A. (2012). miR-200c sensitizes breast cancer cells to doxorubicin treatment by decreasing TrkB and Bmi1 expression. PLoS ONE.

[73] Chen Y., Ke G., Han D., Liang S., Yang G., Wu X. (2014). MicroRNA-181a enhances the chemoresistance of human cervical squamous cell carcinoma to cisplatin by targeting PRKCD. Exp. Cell Res..

[74] Fan Z., Cui H., Yu H., Ji Q., Kang L., Han B., Wang J., Dong Q., Li Y., Yan Z. (2016). MiR-125a promotes paclitaxel sensitivity in cervical cancer through altering STAT3 expression. Oncogenesis.

[75] Zhu Z., Wang C.-P., Zhang Y.-F., Nie L. (2014). MicroRNA-100 resensitizes resistant chondrosarcoma cells to cisplatin through direct targeting of mTOR. Asian Pac. J. Cancer Prev..

[76] Hu J., Xu Y., Cai S. (2015). Specific microRNAs as novel biomarkers for combination chemotherapy resistance detection of colon adenocarcinoma. Eur. J. Med. Res..

[77] Li X., Zhao H., Zhou X., Song L. (2015). Inhibition of lactate dehydrogenase A by microRNA-34a resensitizes colon cancer cells to 5-fluorouracil. Mol. Med. Rep..

[78] He J., Xie G., Tong J., Peng Y., Huang H., Li J., Wang N., Liang H. (2014). Overexpression of microRNA-122 re-sensitizes 5-FU-resistant colon cancer cells to 5-FU through the inhibition of PKM2 in vitro and in vivo. Cell Biochem. Biophys..

[79] Tan S., Shi H., Ba M., Lin S., Tang H., Zeng X., Zhang X. (2016). miR-409-3p sensitizes colon cancer cells to oxaliplatin by inhibiting Beclin-1-mediated autophagy. Int. J. Mol. Med..

[80] Chai J., Dong W., Xie C., Wang L., Han D.-L., Wang S., Guo H.-L., Zhang Z.-L. (2015). MicroRNA-494 sensitizes colon cancer cells to fluorouracil through regulation of DPYD. IUBMB Life.

[81] Chen J., Chen Y., Chen Z. (2013). MiR-125a/b regulates the activation of cancer stem cells in paclitaxel-resistant colon cancer. Cancer Investig..

[82] Li P.-L., Zhang X., Wang L.-L., Du L.-T., Yang Y.-M., Li J., Wang C.-X. (2015). MicroRNA-218 is a prognostic indicator in colorectal cancer and enhances 5-fluorouracil-induced apoptosis by targeting BIRC5. Carcinogenesis.

[83] Liu Y., Gao S., Chen X., Liu M., Mao C., Fang X. (2016). Overexpression of miR-203 sensitizes paclitaxel (Taxol)-resistant colorectal cancer cells through targeting the salt-inducible kinase 2 (SIK2). Tumour Biol..

[84] Li T., Gao F., Zhang X.-P. (2015). miR-203 enhances chemosensitivity to 5-fluorouracil by targeting thymidylate synthase in colorectal cancer. Oncol. Rep..

[85] Hu J., Cai G., Xu Y., Cai S. (2016). The Plasma microRNA miR-1914* and -1915 Suppresses Chemoresistant in Colorectal Cancer Patients by Down-regulating NFIX. Curr. Mol. Med..

[86] Wu H., Liang Y., Shen L., Shen L. (2016). MicroRNA-204 modulates colorectal cancer cell sensitivity in response to 5-fluorouracil-based treatment by targeting high mobility group protein A2. Biol. Open.

[87] Liu H., Yin Y., Hu Y., Feng Y., Bian Z., Yao S., Li M., You Q., Huang Z. (2016). miR-139-5p sensitizes colorectal cancer cells to 5-fluorouracil by targeting NOTCH-1. Pathol. Res. Pract..

[88] Eyking A., Reis H., Frank M., Gerken G., Schmid K.W., Cario E. (2016). MiR-205 and MiR-373 Are Associated with Aggressive Human Mucinous Colorectal Cancer. PLoS ONE.

[89] Zhang Y., Hu X., Miao X., Zhu K., Cui S., Meng Q., Sun J., Wang T. (2016). MicroRNA-425-5p regulates chemoresistance in colorectal cancer cells via regulation of Programmed Cell Death 10. J. Cell. Mol. Med..

[90] Dong S.-J., Cai X.-J., Li S.-J. (2016). The Clinical Significance of MiR-429 as a Predictive Biomarker in Colorectal Cancer Patients Receiving 5-Fluorouracil Treatment. Med. Sci. Monit..

[91] Siemens H., Jackstadt R., Kaller M., Hermeking H. (2013). Repression of c-Kit by p53 is mediated by miR-34 and is associated with reduced chemoresistance, migration and stemness. Oncotarget.

[92] To K.K.W., Leung W.W., Ng S.S.M. (2015). Exploiting a novel miR-519c-HuR-ABCG2 regulatory pathway to overcome chemoresistance in colorectal cancer. Exp. Cell Res..

[93] Zhang Y., Geng L., Talmon G., Wang J. (2015). MicroRNA-520g confers drug resistance by regulating p21 expression in colorectal cancer. J. Biol. Chem..

[94] Li X., Li X., Liao D., Wang X., Wu Z., Nie J., Bai M., Fu X., Mei Q., Han W. (2015). Elevated microRNA-23a Expression Enhances the Chemoresistance of Colorectal Cancer Cells with Microsatellite Instability to 5-Fluorouracil by Directly Targeting ABCF1. Curr. Protein Pept. Sci..

[95] Kim S.-A., Kim I., Yoon S.K., Lee E.K., Kuh H.-J. (2015). Indirect modulation of sensitivity to 5-fluorouracil by microRNA-96 in human colorectal cancer cells. Arch. Pharm. Res..

[96] Zhang Y., Talmon G., Wang J. (2015). MicroRNA-587 antagonizes 5-FU-induced apoptosis and confers drug resistance by regulating PPP2R1B expression in colorectal cancer. Cell Death Dis..

[97] Ran X., Yang J., Liu C., Zhou P., Xiao L., Zhang K. (2015). MiR-218 inhibits HMGB1-mediated autophagy in endometrial carcinoma cells during chemotherapy. Int. J. Clin. Exp. Pathol..

[98] Iida K., Fukushi J.-I., Matsumoto Y., Oda Y., Takahashi Y., Fujiwara T., Fujiwara-Okada Y., Hatano M., Nabashima A., Kamura S. (2013). miR-125b develops chemoresistance in Ewing sarcoma/primitive neuroectodermal tumor. Cancer Cell Int..

[99] Zhan M., Zhao X., Wang H., Chen W., Xu S., Wang W., Shen H., Huang S., Wang J. (2016). miR-145 sensitizes gallbladder cancer to cisplatin by regulating multidrug resistance associated protein 1. Tumour Biol..

[100] Cao W., Wei W., Zhan Z., Xie Y., Xiao Q. (2016). MiR-1284 modulates multidrug resistance of gastric cancer cells by targeting EIF4A1. Oncol. Rep..

[101] Zhou N., Qu Y., Xu C., Tang Y. (2016). Upregulation of microRNA-375 increases the cisplatin-sensitivity of human gastric cancer cells by regulating ERBB2. Exp. Ther. Med..

[102] An Y., Zhang Z., Shang Y., Jiang X., Dong J., Yu P., Nie Y., Zhao Q. (2015). miR-23b-3p regulates the chemoresistance of gastric cancer cells by targeting ATG12 and HMGB2. Cell Death Dis..

[103] Zhu M., Zhou X., Du Y., Huang Z., Zhu J., Xu J., Cheng G., Shu Y., Liu P., Zhu W. (2016). miR-20a induces cisplatin resistance of a human gastric cancer cell line via targeting CYLD. Mol. Med. Rep..

[104] Wu H., Huang M., Lu M., Zhu W., Shu Y., Cao P., Liu P. (2013). Regulation of microtubule-associated protein tau (MAPT) by miR-34c-5p determines the chemosensitivity of gastric cancer to paclitaxel. Cancer Chemother. Pharmacol..

[105] Wang F., Song X., Li X., Xin J., Wang S., Yang W., Wang J., Wu K., Chen X., Liang J. (2013). Noninvasive visualization of microRNA-16 in the chemoresistance of gastric cancer using a dual reporter gene imaging system. PLoS ONE.

[106] Munoz J.L., Rodriguez-Cruz V., Rameshwar P. (2015). High expression of miR-9 in CD133(+) glioblastoma cells in chemoresistance to temozolomide. J. Cancer Stem Cell Res..

[107] Zhou D., Wan Y., Xie D., Wang Y., Wei J., Yan Q., Lu P., Mo L., Xie J., Yang S., Qi X. (2015). DNMT1 mediates chemosensitivity by reducing methylation of miRNA-20a promoter in glioma cells. Exp. Mol. Med..

[108] Giunti L., da Ros M., Vinci S., Gelmini S., Iorio A.L., Buccoliero A.M., Cardellicchio S., Castiglione F., Genitori L., de Martino M. (2015). Anti-miR21 oligonucleotide enhances chemosensitivity of T98G cell line to doxorubicin by inducing apoptosis. Am. J. Cancer Res..

[109] Chen X., Zhang Y., Shi Y., Lian H., Tu H., Han S., Peng B., Liu W., He X. (2015). MiR-873 acts as a novel sensitizer of glioma cells to cisplatin by targeting Bcl-2. Int. J. Oncol..

[110] Lee D., Sun S., Zhang X.Q., Zhang P.D., Ho A.S.W., Kiang K.M.Y., Fung C.F., Lui W.M., Leung G.K.K. (2015). MicroRNA-210 and Endoplasmic Reticulum Chaperones in the Regulation of Chemoresistance in Glioblastoma. J. Cancer.

[111] Stojcheva N., Schechtmann G., Sass S., Roth P., Florea A.-M., Stefanski A., Stühler K., Wolter M., Müller N.S., Theis F.J. (2016). MicroRNA-138 promotes acquired alkylator resistance in glioblastoma by targeting the Bcl-2-interacting mediator BIM. Oncotarget.

[112] Haemmig S., Baumgartner U., Glück A., Zbinden S., Tschan M.P., Kappeler A., Mariani L., Vajtai I., Vassella E. (2014). miR-125b controls apoptosis and temozolomide resistance by targeting TNFAIP3 and NKIRAS2 in glioblastomas. Cell Death Dis..

[113] Liao H., Bai Y., Qiu S., Zheng L., Huang L., Liu T., Wang X., Liu Y., Xu N., Yan X. (2015). MiR-203 downregulation is responsible for chemoresistance in human glioblastoma by promoting epithelial-mesenchymal transition via SNAI2. Oncotarget.

[114] Guo Y., Yan K., Fang J., Qu Q., Zhou M., Chen F. (2013). Let-7b expression determines response to chemotherapy through the regulation of cyclin D1 in glioblastoma. J. Exp. Clin. Cancer Res. CR.

[115] Wang J., Sai K., Chen F., Chen Z. (2013). miR-181b modulates glioma cell sensitivity to temozolomide by targeting MEK1. Cancer Chemother. Pharmacol..

[116] Shi Z., Chen Q., Li C., Wang L., Qian X., Jiang C., Liu X., Wang X., Li H., Kang C. (2014). MiR-124 governs glioma growth and angiogenesis and enhances chemosensitivity by targeting R-Ras and N-Ras. Neuro-Oncol..

[117] Berthois Y., Delfino C., Metellus P., Fina F., Nanni-Metellus I., Al Aswy H., Pirisi V., Ouafik L., Boudouresque F. (2014). Differential expression of miR200a-3p and miR21 in grade II-III and grade IV gliomas: Evidence that miR200a-3p is regulated by O^6^-methylguanine methyltransferase and promotes temozolomide responsiveness. Cancer Biol. Ther..

[118] Chen W., Yang Y., Chen B., Lu P., Zhan L., Yu Q., Cao K., Li Q. (2014). MiR-136 targets E2F1 to reverse cisplatin chemosensitivity in glioma cells. J. Neurooncol..

[119] Bourguignon L.Y.W., Wong G., Shiina M. (2016). Up-regulation of Histone Methyltransferase, DOT1L, by Matrix Hyaluronan Promotes MicroRNA-10 Expression Leading to Tumor Cell Invasion and Chemoresistance in Cancer Stem Cells from Head and Neck Squamous Cell Carcinoma. J. Biol. Chem..

[120] Zhang K., Chen J., Chen D., Huang J., Feng B., Han S., Chen Y., Song H., De W., Zhu Z. (2014). Aurora-A promotes chemoresistance in hepatocelluar carcinoma by targeting NF-kappaB/microRNA-21/PTEN signaling pathway. Oncotarget.

[121] Yang F., Li Q., Gong Z., Zhou L., You N., Wang S., Li X., Li J., An J., Wang D., He Y., Dou K. (2014). MicroRNA-34a targets Bcl-2 and sensitizes human hepatocellular carcinoma cells to sorafenib treatment. Technol. Cancer Res. Treat..

[122] Zhao N., Wang R., Zhou L., Zhu Y., Gong J., Zhuang S.-M. (2014). MicroRNA-26b suppresses the NF-κB signaling and enhances the chemosensitivity of hepatocellular carcinoma cells by targeting TAK1 and TAB3. Mol. Cancer.

[123] Wang R., Li Y., Hou Y., Yang Q., Chen S., Wang X., Wang Z., Yang Y., Chen C., Wang Z. (2015). The PDGF-D/miR-106a/Twist1 pathway orchestrates epithelial-mesenchymal transition in gemcitabine resistance hepatoma cells. Oncotarget.

[124] Xu Y., An Y., Wang Y., Zhang C., Zhang H., Huang C., Jiang H., Wang X., Li X. (2013). miR-101 inhibits autophagy and enhances cisplatin-induced apoptosis in hepatocellular carcinoma cells. Oncol. Rep..

[125] Jiang J.-X., Gao S., Pan Y.-Z., Yu C., Sun C.-Y. (2014). Overexpression of microRNA-125b sensitizes human hepatocellular carcinoma cells to 5-fluorouracil through inhibition of glycolysis by targeting hexokinase II. Mol. Med. Rep..

[126] Ju B.-L., Chen Y.-B., Zhang W.-Y., Yu C.-H., Zhu D.-Q., Jin J. (2015). miR-145 regulates chemoresistance in hepatocellular carcinoma via epithelial mesenchymal transition. Cell. Mol. Biol. Noisy.

[127] Shi L., Wu L., Chen Z., Yang J., Chen X., Yu F., Zheng F., Lin X. (2015). MiR-141 Activates Nrf2-Dependent Antioxidant Pathway via Down-Regulating the Expression of Keap1 Conferring the Resistance of Hepatocellular Carcinoma Cells to 5-Fluorouracil. Cell. Physiol. Biochem..

[128] Kishikawa T., Otsuka M., Tan P.S., Ohno M., Sun X., Yoshikawa T., Shibata C., Takata A., Kojima K., Takehana K. (2015). Decreased miR122 in hepatocellular carcinoma leads to chemoresistance with increased arginine. Oncotarget.

[129] Shi L., Chen Z.-G., Wu L.-L., Zheng J.-J., Yang J.-R., Chen X.-F., Chen Z.-Q., Liu C.-L., Chi S.-Y., Zheng J.-Y. (2014). miR-340 reverses cisplatin resistance of hepatocellular carcinoma cell lines by targeting Nrf2-dependent antioxidant pathway. Asian Pac. J. Cancer Prev..

[130] Qin J., Luo M., Qian H., Chen W. (2014). Upregulated miR-182 increases drug resistance in cisplatin-treated HCC cell by regulating TP53INP1. Gene.

[131] Wang L., Wang Y.M., Xu S., Wang W.G., Chen Y., Mao J.Y., Tian B.L. (2015). MicroRNA-215 is upregulated by treatment with Adriamycin and leads to the chemoresistance of hepatocellular carcinoma cells and tissues. Mol. Med. Rep..

[132] Ho T.-T., He X., Mo Y.-Y., Beck W.T. (2016). Transient resistance to DNA damaging agents is associated with expression of microRNAs-135b and -196b in human leukemia cell lines. Int. J. Biochem. Mol. Biol..

[133] Weng H., Huang H., Dong B., Zhao P., Zhou H., Qu L. (2014). Inhibition of miR-17 and miR-20a by oridonin triggers apoptosis and reverses chemoresistance by derepressing BIM-S. Cancer Res..

[134] Seca H., Lima R.T., Lopes-Rodrigues V., Guimaraes J.E., Almeida G.M., Vasconcelos M.H. (2013). Targeting miR-21 induces autophagy and chemosensitivity of leukemia cells. Curr. Drug Targets.

[135] Yan Z.-X., Zheng Z., Xue W., Zhao M.-Z., Fei X.-C., Wu L.-L., Huang L.-M., Leboeuf C., Janin A., Wang L. (2015). MicroRNA181a Is Overexpressed in T-Cell Leukemia/Lymphoma and Related to Chemoresistance. BioMed Res. Int..

[136] Zhao L., Li Y., Song X., Zhou H., Li N., Miao Y., Jia L. (2016). Upregulation of miR-181c inhibits chemoresistance by targeting ST8SIA4 in chronic myelocytic leukemia. Oncotarget.

[137] Chen Y., Jacamo R., Konopleva M., Garzon R., Croce C., Andreeff M. (2013). CXCR4 downregulation of let-7a drives chemoresistance in acute myeloid leukemia. J. Clin. Investig..

[138] Zhan M., Qu Q., Wang G., Zhou H. (2013). Let-7c sensitizes acquired cisplatin-resistant A549 cells by targeting ABCC2 and Bcl-XL. Die Pharm..

[139] Li W., Wang W., Ding M., Zheng X., Ma S., Wang X. (2016). MiR-1244 sensitizes the resistance of non-small cell lung cancer A549 cell to cisplatin. Cancer Cell Int..

[140] Wu L., Pu X., Wang Q., Cao J., Xu F., Xu L.I., Li K. (2016). miR-96 induces cisplatin chemoresistance in non-small cell lung cancer cells by downregulating SAMD9. Oncol. Lett..

[141] Zhang Z., Zhang L., Yin Z.-Y., Fan X.-L., Hu B., Wang L.-Q., Zhang D. (2014). miR-107 regulates cisplatin chemosensitivity of A549 non small cell lung cancer cell line by targeting cyclin dependent kinase 8. Int. J. Clin. Exp. Pathol..

[142] Chen X., Jiang Y., Huang Z., Li D., Chen X., Cao M., Meng Q., Pang H., Sun L., Zhao Y. (2016). miRNA-378 reverses chemoresistance to cisplatin in lung adenocarcinoma cells by targeting secreted clusterin. Sci. Rep..

[143] Zhang F., Li Y., Wu H., Qi K., You J., Li X., Zu L., Pan Z., Wang Y., Li Y. (2014). MiR-192 confers cisplatin resistance by targeting Bim in lung cancer. Chin. J. Lung Cancer.

[144] Lei L., Huang Y., Gong W. (2013). miR-205 promotes the growth, metastasis and chemoresistance of NSCLC cells by targeting PTEN. Oncol. Rep..

[145] Yang Z., Fang S., Di Y., Ying W., Tan Y., Gu W. (2015). Modulation of NF-κB/miR-21/PTEN pathway sensitizes non-small cell lung cancer to cisplatin. PLoS ONE.

[146] Pan B., Chen Y., Song H., Xu Y., Wang R., Chen L. (2015). Mir-24-3p downregulation contributes to VP16-DDP resistance in small-cell lung cancer by targeting ATG4A. Oncotarget.

[147] Zheng D., Dai Y., Wang S., Xing X. (2015). MicroRNA-299-3p promotes the sensibility of lung cancer to doxorubicin through directly targeting ABCE1. Int. J. Clin. Exp. Pathol..

[148] Li J., Wang Y., Song Y., Fu Z., Yu W. (2014). miR-27a regulates cisplatin resistance and metastasis by targeting RKIP in human lung adenocarcinoma cells. Mol. Cancer.

[149] Xu X., Wells A., Padilla M.T., Kato K., Kim K.C., Lin Y. (2014). A signaling pathway consisting of miR-551b, catalase and MUC1 contributes to acquired apoptosis resistance and chemoresistance. Carcinogenesis.

[150] Xiao F., Bai Y., Chen Z., Li Y., Luo L., Huang J., Yang J., Liao H., Guo L. (2014). Downregulation of HOXA1 gene affects small cell lung cancer cell survival and chemoresistance under the regulation of miR-100. Eur. J. Cancer.

[151] Wang Q., Chen W., Bai L., Chen W., Padilla M.T., Lin A.S., Shi S., Wang X., Lin Y. (2014). Receptor-interacting protein 1 increases chemoresistance by maintaining inhibitor of apoptosis protein levels and reducing reactive oxygen species through a microRNA-146a-mediated catalase pathway. J. Biol. Chem..

[152] Ning F., Wang F., Li M., Yu Z.-S., Hao Y., Chen S. (2014). MicroRNA-182 modulates chemosensitivity of human non-small cell lung cancer to cisplatin by targeting PDCD4. Diagn. Pathol..

[153] Huang J.-Y., Cui S.-Y., Chen Y.-T., Song H.-Z., Huang G.-C., Feng B., Sun M., De W., Wang R., Chen L.-B. (2013). MicroRNA-650 was a prognostic factor in human lung adenocarcinoma and confers the docetaxel chemoresistance of lung adenocarcinoma cells via regulating Bcl-2/Bax expression. PLoS ONE.

[154] Wang H., Zhu L.-J., Yang Y.-C., Wang Z.-X., Wang R. (2014). MiR-224 promotes the chemoresistance of human lung adenocarcinoma cells to cisplatin via regulating G₁/S transition and apoptosis by targeting p21(WAF1/CIP1). Br. J. Cancer.

[155] Chen D., Huang J., Zhang K., Pan B., Chen J., De W., Wang R., Chen L. (2014). MicroRNA-451 induces epithelial-mesenchymal transition in docetaxel-resistant lung adenocarcinoma cells by targeting proto-oncogene c-Myc. Eur. J. Cancer Oxf. Engl. 1990.

[156] Zhao Z., Zhang L., Yao Q., Tao Z. (2015). miR-15b regulates cisplatin resistance and metastasis by targeting PEBP4 in human lung adenocarcinoma cells. Cancer Gene Ther..

[157] Sui C., Meng F., Li Y., Jiang Y. (2015). miR-148b reverses cisplatin-resistance in non-small cell cancer cells via negatively regulating DNA (cytosine-5)-methyltransferase 1(DNMT1) expression. J. Transl. Med..

[158] Zarogoulidis P., Petanidis S., Kioseoglou E., Domvri K., Anestakis D., Zarogoulidis K. (2015). MiR-205 and miR-218 expression is associated with carboplatin chemoresistance and regulation of apoptosis via Mcl-1 and Survivin in lung cancer cells. Cell. Signal..

[159] Liang N., Zhou X., Zhao M., Zhao D., Zhu Z., Li S., Yang H. (2015). Down-regulation of microRNA-26b modulates non-small cell lung cancer cells chemoresistance and migration through the association of PTEN. Acta Biochim. Biophys. Sin..

[160] Cao J., He Y., Liu H.-Q., Wang S.-B., Zhao B.-C., Cheng Y.-S. (2015). MicroRNA 192 regulates chemo-resistance of lung adenocarcinoma for gemcitabine and cisplatin combined therapy by targeting Bcl-2. Int. J. Clin. Exp. Med..

[161] Fujita Y., Yagishita S., Hagiwara K., Yoshioka Y., Kosaka N., Takeshita F., Fujiwara T., Tsuta K., Nokihara H., Tamura T. (2015). The clinical relevance of the miR-197/CKS1B/STAT3-mediated PD-L1 network in chemoresistant non-small-cell lung cancer. Mol. Ther. J. Am. Soc. Gene Ther..

[162] Liu H., Wu X., Huang J., Peng J., Guo L. (2015). miR-7 modulates chemoresistance of small cell lung cancer by repressing MRP1/ABCC1. Int. J. Exp. Pathol..

[163] Wang Q., Shi S., He W., Padilla M.T., Zhang L., Wang X., Zhang B., Lin Y. (2014). Retaining MKP1 expression and attenuating JNK-mediated apoptosis by RIP1 for cisplatin resistance through miR-940 inhibition. Oncotarget.

[164] Pan B., Feng B., Chen Y., Huang G., Wang R., Chen L., Song H. (2015). MiR-200b regulates autophagy associated with chemoresistance in human lung adenocarcinoma. Oncotarget.

[165] Shan W., Zhang X., Li M., Deng F., Zhang J. (2016). Over expression of miR-200c suppresses invasion and restores methotrexate sensitivity in lung cancer A549 cells. Gene.

[166] Bai Y., Sun Y., Peng J., Liao H., Gao H., Guo Y., Guo L. (2014). Overexpression of secretagogin inhibits cell apoptosis and induces chemoresistance in small cell lung cancer under the regulation of miR-494. Oncotarget.

[167] Al-Harbi S., Choudhary G.S., Ebron J.S., Hill B.T., Vivekanathan N., Ting A.H., Radivoyevitch T., Smith M.R., Shukla G.C., Almasan A. (2015). miR-377-dependent BCL-xL regulation drives chemotherapeutic resistance in B-cell lymphoid malignancies. Mol. Cancer.

[168] Yuan W.X., Gui Y.X., Na W.N., Chao J., Yang X. (2016). Circulating microRNA-125b and microRNA-130a expression profiles predict chemoresistance to R-CHOP in diffuse large B-cell lymphoma patients. Oncol. Lett..

[169] Yang G.-D., Huang T.-J., Peng L.-X., Yang C.-F., Liu R.-Y., Huang H.-B., Chu Q.-Q., Yang H.-J., Huang J.-L., Zhu Z.-Y. (2013). Epstein-Barr Virus_Encoded LMP1 upregulates microRNA-21 to promote the resistance of nasopharyngeal carcinoma cells to cisplatin-induced Apoptosis by suppressing PDCD4 and Fas-L. PLoS ONE.

[170] Peng X., Cao P., He D., Han S., Zhou J., Tan G., Li W., Yu F., Yu J., Li Z. (2014). MiR-634 sensitizes nasopharyngeal carcinoma cells to paclitaxel and inhibits cell growth both in vitro and in vivo. Int. J. Clin. Exp. Pathol..

[171] Phatak P., Byrnes K.A., Mansour D., Liu L., Cao S., Li R., Rao J.N., Turner D.J., Wang J.-Y., Donahue J.M. (2016). Overexpression of miR-214-3p in esophageal squamous cancer cells enhances sensitivity to cisplatin by targeting survivin directly and indirectly through CUG-BP1. Oncogene.

[172] Komatsu S., Ichikawa D., Kawaguchi T., Miyamae M., Okajima W., Ohashi T., Imamura T., Kiuchi J., Konishi H., Shiozaki A. (2016). Circulating miR-21 as an independent predictive biomarker for chemoresistance in esophageal squamous cell carcinoma. Am. J. Cancer Res..

[173] Meng F., Qian L., Lv L., Ding B., Zhou G., Cheng X., Niu S., Liang Y. (2016). miR-193a-3p regulation of chemoradiation resistance in oesophageal cancer cells via the PSEN1 gene. Gene.

[174] Tanaka K., Miyata H., Sugimura K., Fukuda S., Kanemura T., Yamashita K., Miyazaki Y., Takahashi T., Kurokawa Y., Yamasaki M. (2015). miR-27 is associated with chemoresistance in esophageal cancer through transformation of normal fibroblasts to cancer-associated fibroblasts. Carcinogenesis.

[175] Wang Y., Zhao Y., Herbst A., Kalinski T., Qin J., Wang X., Jiang Z., Benedix F., Franke S., Wartman T. (2016). miR-221 Mediates Chemoresistance of Esophageal Adenocarcinoma by Direct Targeting of DKK2 Expression. Ann. Surg..

[176] Liu M., Wang J., Huang H., Hou J., Zhang B., Wang A. (2013). miR-181a-Twist1 pathway in the chemoresistance of tongue squamous cell carcinoma. Biochem. Biophys. Res. Commun..

[177] Peng F., Zhang H., Du Y., Tan P. (2015). miR-23a promotes cisplatin chemoresistance and protects against cisplatin-induced apoptosis in tongue squamous cell carcinoma cells through Twist. Oncol. Rep..

[178] Zhou J., Wu S., Chen Y., Zhao J., Zhang K., Wang J., Chen S. (2015). microRNA-143 is associated with the survival of ALDH1+CD133+ osteosarcoma cells and the chemoresistance of osteosarcoma. Exp. Biol. Med..

[179] Chang Z., Huo L., Li K., Wu Y., Hu Z. (2014). Blocked autophagy by miR-101 enhances osteosarcoma cell chemosensitivity in vitro. Scientific World J..

[180] Di Fiore R., Drago-Ferrante R., Pentimalli F., Di Marzo D., Forte I.M., D’Anneo A., Carlisi D., De Blasio A., Giuliano M., Tesoriere G. (2014). MicroRNA-29b-1 impairs in vitro cell proliferation, self‑renewal and chemoresistance of human osteosarcoma 3AB-OS cancer stem cells. Int. J. Oncol..

[181] Zhou Y., Huang Z., Wu S., Zang X., Liu M., Shi J. (2014). miR-33a is up-regulated in chemoresistant osteosarcoma and promotes osteosarcoma cell resistance to cisplatin by down-regulating TWIST. J. Exp. Clin. Cancer Res..

[182] Xu M., Jin H., Xu C.-X., Bi W.-Z., Wang Y. (2014). MiR-34c inhibits osteosarcoma metastasis and chemoresistance. Med. Oncol. Northwood Lond. Engl..

[183] Zhang Y., Duan G., Feng S. (2015). MicroRNA-301a modulates doxorubicin resistance in osteosarcoma cells by targeting AMP-activated protein kinase alpha 1. Biochem. Biophys. Res. Commun..

[184] Li X., Wang S., Chen Y., Liu G., Yang X. (2014). miR-22 targets the 3’ UTR of HMGB1 and inhibits the HMGB1-associated autophagy in osteosarcoma cells during chemotherapy. Tumour Biol..

[185] Xu M., Jin H., Xu C.-X., Sun B., Mao Z., Bi W.-Z., Wang Y. (2014). miR-382 inhibits tumor growth and enhance chemosensitivity in osteosarcoma. Oncotarget.

[186] Jacques C., Calleja L. R., Baud’huin M., Quillard T., Heymann D., Lamoureux F., Ory B. (2016). miRNA-193a-5p repression of p73 controls Cisplatin chemoresistance in primary bone tumors. Oncotarget.

[187] Zhao H., Liu S., Wang G., Wu X., Ding Y., Guo G., Jiang J., Cui S. (2015). Expression of miR-136 is associated with the primary cisplatin resistance of human epithelial ovarian cancer. Oncol. Rep..

[188] Sestito R., Cianfrocca R., Rosanò L., Tocci P., Semprucci E., Di Castro V., Caprara V., Ferrandina G., Sacconi A., Blandino G. (2016). miR-30a inhibits endothelin A receptor and chemoresistance in ovarian carcinoma. Oncotarget.

[189] Zhan Y., Xiang F., Wu R., Xu J., Ni Z., Jiang J., Kang X. (2015). MiRNA-149 modulates chemosensitivity of ovarian cancer A2780 cells to paclitaxel by targeting MyD88. J. Ovarian Res..

[190] Li X., Pan Q., Wan X., Mao Y., Lu W., Xie X., Cheng X. (2015). Methylation-associated Has-miR-9 deregulation in paclitaxel- resistant epithelial ovarian carcinoma. BMC Cancer.

[191] Pink R.C., Samuel P., Massa D., Caley D.P., Brooks S.A., Carter D.R.F. (2015). The passenger strand, miR-21-3p, plays a role in mediating cisplatin resistance in ovarian cancer cells. Gynecol. Oncol..

[192] Samuel P., Pink R.C., Caley D.P., Currie J.M.S., Brooks S.A., Carter D.R.F. (2016). Over-expression of miR-31 or loss of KCNMA1 leads to increased cisplatin resistance in ovarian cancer cells. Tumour Biol..

[193] Hassan M.K., Watari H., Mitamura T., Mohamed Z., El-Khamisy S.F., Ohba Y., Sakuragi N. (2015). P18/Stathmin1 is regulated by miR-31 in ovarian cancer in response to taxane. Oncoscience.

[194] Sugio A., Iwasaki M., Habata S., Mariya T., Suzuki M., Osogami H., Tamate M., Tanaka R., Saito T. (2014). BAG3 upregulates Mcl-1 through downregulation of miR-29b to induce anticancer drug resistance in ovarian cancer. Gynecol. Oncol..

[195] Liu N., Zeng J., Zhang X., Yang Q., Liao D., Chen G., Wang Y. (2014). Involvement of miR-200a in chemosensitivity regulation of ovarian cancer. Zhonghua Yi Xue Za Zhi.

[196] Liu G., Yang D., Rupaimoole R., Pecot C.V., Sun Y., Mangala L.S., Li X., Ji P., Cogdell D., Hu L. (2015). Augmentation of response to chemotherapy by microRNA-506 through regulation of RAD51 in serous ovarian cancers. J. Natl. Cancer Inst..

[197] Weiner-Gorzel K., Dempsey E., Milewska M., McGoldrick A., Toh V., Walsh A., Lindsay S., Gubbins L., Cannon A., Sharpe D. (2015). Overexpression of the microRNA miR-433 promotes resistance to paclitaxel through the induction of cellular senescence in ovarian cancer cells. Cancer Med..

[198] Zhu X., Shen H., Yin X., Long L., Xie C., Liu Y., Hui L., Lin X., Fang Y., Cao Y. (2016). miR-186 regulation of Twist1 and ovarian cancer sensitivity to cisplatin. Oncogene.

[199] Zhou Y., Wang M., Wu J., Jie Z., Chang S., Shuang T. (2015). The clinicopathological significance of miR-1307 in chemotherapy resistant epithelial ovarian cancer. J. Ovarian Res..

[200] Zhao H., Bi T., Qu Z., Jiang J., Cui S., Wang Y. (2014). Expression of miR-224-5p is associated with the original cisplatin resistance of ovarian papillary serous carcinoma. Oncol. Rep..

[201] Li N., Yang L., Wang H., Yi T., Jia X., Chen C., Xu P. (2015). MiR-130a and MiR-374a Function as Novel Regulators of Cisplatin Resistance in Human Ovarian Cancer A2780 Cells. PLoS ONE.

[202] Rao Y., Shi H., Ji M., Chen C. (2013). MiR-106a targets Mcl-1 to suppress cisplatin resistance of ovarian cancer A2780 cells. J. Huazhong Univ. Sci. Technol. Med. Sci..

[203] Huh J.H., Kim T.H., Kim K., Song J.-A., Jung Y.J., Jeong J.-Y., Lee M.J., Kim Y.K., Lee D.H., An H.J. (2013). Dysregulation of miR-106a and miR-591 confers paclitaxel resistance to ovarian cancer. Br. J. Cancer.

[204] Zhao H., Yu X., Ding Y., Zhao J., Wang G., Wu X., Jiang J., Peng C., Guo G.Z., Cui S. (2016). MiR-770-5p inhibits cisplatin chemoresistance in human ovarian cancer by targeting ERCC2. Oncotarget.

[205] Au Yeung C.L., Co N.-N., Tsuruga T., Yeung T.-L., Kwan S.-Y., Leung C.S., Li Y., Lu E.S., Kwan K., Wong K.-K. (2016). Exosomal transfer of stroma-derived miR21 confers paclitaxel resistance in ovarian cancer cells through targeting APAF1. Nat. Commun..

[206] Liu M.X., Siu M.K.Y., Liu S.S., Yam J.W.P., Ngan H.Y.S., Chan D.W. (2014). Epigenetic silencing of microRNA-199b-5p is associated with acquired chemoresistance via activation of JAG1-Notch1 signaling in ovarian cancer. Oncotarget.

[207] Zhu X., Li Y., Xie C., Yin X., Liu Y., Cao Y., Fang Y., Lin X., Xu Y., Xu W. (2014). miR-145 sensitizes ovarian cancer cells to paclitaxel by targeting Sp1 and Cdk6. Int. J. Cancer.

[208] Cai J., Yang C., Yang Q., Ding H., Jia J., Guo J., Wang J., Wang Z. (2013). Deregulation of let-7e in epithelial ovarian cancer promotes the development of resistance to cisplatin. Oncogenesis.

[209] He J., Yu J.-J., Xu Q., Wang L., Zheng J.Z., Liu L.-Z., Jiang B.-H. (2015). Downregulation of ATG14 by EGR1-MIR152 sensitizes ovarian cancer cells to cisplatin-induced apoptosis by inhibiting cyto-protective autophagy. Autophagy.

[210] Li B., Chen H., Wu N., Zhang W.-J., Shang L.-X. (2014). Deregulation of miR-128 in ovarian cancer promotes cisplatin resistance. Int. J. Gynecol. Cancer.

[211] Vecchione A., Belletti B., Lovat F., Volinia S., Chiappetta G., Giglio S., Sonego M., Cirombella R., Onesti E.C., Pellegrini P. (2013). A microRNA signature defines chemoresistance in ovarian cancer through modulation of angiogenesis. Proc. Natl. Acad. Sci. USA.

[212] Park Y.T., Jeong J.-Y., Lee M.-J., Kim K.-I., Kim T.-H., Kwon Y., Lee C., Kim O.J., An H.-J. (2013). MicroRNAs overexpressed in ovarian ALDH1-positive cells are associated with chemoresistance. J. Ovarian Res..

[213] Frederick P.J., Green H.N., Huang J.S., Egger M.E., Frieboes H.B., Grizzle W.E., McNally L.R. (2013). Chemoresistance in ovarian cancer linked to expression of microRNAs. Biotech. Histochem..

[214] Wang Y.-Q., Guo R.-D., Guo R.-M., Sheng W., Yin L.-R. (2013). MicroRNA-182 promotes cell growth, invasion, and chemoresistance by targeting programmed cell death 4 (PDCD4) in human ovarian carcinomas. J. Cell. Biochem..

[215] Ma C., Huang T., Ding Y.-C., Yu W., Wang Q., Meng B., Luo S.-X. (2015). MicroRNA-200c overexpression inhibits chemoresistance, invasion and colony formation of human pancreatic cancer stem cells. Int. J. Clin. Exp. Pathol..

[216] Liang C., Wang Z., Li Y.-Y., Yu B.-H., Zhang F., Li H.-Y. (2015). miR-33a suppresses the nuclear translocation of β-catenin to enhance gemcitabine sensitivity in human pancreatic cancer cells. Tumour Biol..

[217] Cioffi M., Trabulo S.M., Sanchez-Ripoll Y., Miranda-Lorenzo I., Lonardo E., Dorado J., Reis Vieira C., Ramirez J.C., Hidalgo M., Aicher A. (2015). The miR-17-92 cluster counteracts quiescence and chemoresistance in a distinct subpopulation of pancreatic cancer stem cells. Gut.

[218] Zhao L., Zou D., Wei X., Wang L., Zhang Y., Liu S., Si Y., Zhao H., Wang F., Yu J. (2016). MiRNA-221-3p desensitizes pancreatic cancer cells to 5-fluorouracil by targeting RB1. Tumour Biol..

[219] Hasegawa S., Eguchi H., Nagano H., Konno M., Tomimaru Y., Wada H., Hama N., Kawamoto K., Kobayashi S., Nishida N. (2014). MicroRNA-1246 expression associated with CCNG2-mediated chemoresistance and stemness in pancreatic cancer. Br. J. Cancer.

[220] Takiuchi D., Eguchi H., Nagano H., Iwagami Y., Tomimaru Y., Wada H., Kawamoto K., Kobayashi S., Marubashi S., Tanemura M. (2013). Involvement of microRNA-181b in the gemcitabine resistance of pancreatic cancer cells. Pancreatology.

[221] Liu Y., Li X., Zhu S., Zhang J., Yang M., Qin Q., Deng S., Wang B., Tian K., Liu L. (2015). Ectopic expression of miR-494 inhibited the proliferation, invasion and chemoresistance of pancreatic cancer by regulating SIRT1 and c-Myc. Gene Ther..

[222] Fan P., Liu L., Yin Y., Zhao Z., Zhang Y., Amponsah P.S., Xiao X., Bauer N., Abukiwan A., Nwaeburu C.C. (2016). MicroRNA-101-3p reverses gemcitabine resistance by inhibition of ribonucleotide reductase M1 in pancreatic cancer. Cancer Lett..

[223] Dhayat S.A., Abdeen B., Köhler G., Senninger N., Haier J., Mardin W.A. (2015). MicroRNA-100 and microRNA-21 as markers of survival and chemotherapy response in pancreatic ductal adenocarcinoma UICC stage II. Clin. Epigenetics.

[224] Dhayat S.A., Mardin W.A., Seggewiß J., Ströse A.J., Matuszcak C., Hummel R., Senninger N., Mees S.T., Haier J. (2015). MicroRNA Profiling Implies New Markers of Gemcitabine Chemoresistance in Mutant p53 Pancreatic Ductal Adenocarcinoma. PLoS ONE.

[225] Bhutia Y.D., Hung S.W., Krentz M., Patel D., Lovin D., Manoharan R., Thomson J.M., Govindarajan R. (2013). Differential processing of let-7a precursors influences RRM2 expression and chemosensitivity in pancreatic cancer: Role of LIN-28 and SET oncoprotein. PLoS ONE.

[226] Singh S., Chitkara D., Kumar V., Behrman S.W., Mahato R.I. (2013). miRNA profiling in pancreatic cancer and restoration of chemosensitivity. Cancer Lett..

[227] Li J., Wu H., Li W., Yin L., Guo S., Xu X., Ouyang Y., Zhao Z., Liu S., Tian Y. (2016). Downregulated miR-506 expression facilitates pancreatic cancer progression and chemoresistance via SPHK1/Akt/NF-κB signaling. Oncogene.

[228] Li J., Yang X., Guan H., Mizokami A., Keller E.T., Xu X., Liu X., Tan J., Hu L., Lu Y. (2016). Exosome-derived microRNAs contribute to prostate cancer chemoresistance. Int. J. Oncol..

[229] Kim E.-A., Kim S.-W., Nam J., Sung E.-G., Song I.-H., Kim J.-Y., Kwon T.K., Lee T.-J. (2016). Inhibition of c-FLIPL expression by miRNA-708 increases the sensitivity of renal cancer cells to anti-cancer drugs. Oncotarget.

[230] Peng J., Mo R., Ma J., Fan J. (2015). let-7b and let-7c are determinants of intrinsic chemoresistance in renal cell carcinoma. World J. Surg. Oncol..

[231] Chang I., Mitsui Y., Fukuhara S., Gill A., Wong D.K., Yamamura S., Shahryari V., Tabatabai Z.L., Dahiya R., Shin D.M. (2015). Loss of miR-200c up-regulates CYP1B1 and confers docetaxel resistance in renal cell carcinoma. Oncotarget.

[232] Ayers D., Mestdagh P., Van Maerken T., Vandesompele J. (2015). Identification of miRNAs contributing to neuroblastoma chemoresistance. Comput. Struct. Biotechnol. J..

[233] Valadi H., Ekström K., Bossios A., Sjöstrand M., Lee J.J., Lötvall J.O. (2007). Exosome-mediated transfer of mRNAs and microRNAs is a novel mechanism of genetic exchange between cells. Nat. Cell Biol..

[234] Volders P.-J., Helsens K., Wang X., Menten B., Martens L., Gevaert K., Vandesompele J., Mestdagh P. (2013). LNCipedia: A database for annotated human lncRNA transcript sequences and structures. Nucleic Acids Res..

[235] Volders P.J., Verheggen K., Menschaert G., Vandepoele K., Martens L., Vandesompele J., Mestdagh P. (2015). An update on LNCipedia: A database for annotated human lncRNA sequences. Nucleic Acids Res..

[236] Fang S., Gao H., Tong Y., Yang J., Tang R., Niu Y., Li M., Guo L. (2016). Long noncoding RNA-HOTAIR affects chemoresistance by regulating HOXA1 methylation in small cell lung cancer cells. Lab. Investig..

[237] Liu Z., Sun M., Lu K., Liu J., Zhang M., Wu W., De W., Wang Z., Wang R. (2013). The long noncoding RNA HOTAIR contributes to cisplatin resistance of human lung adenocarcinoma cells via downregualtion of p21(WAF1/CIP1) expression. PLoS ONE.

[238] Özeş A.R., Miller D.F., Özeş O.N., Fang F., Liu Y., Matei D., Huang T., Nephew K.P. (2016). NF-κB-HOTAIR axis links DNA damage response, chemoresistance and cellular senescence in ovarian cancer. Oncogene.

[239] Wang Y., Zhang D., Wu K., Zhao Q., Nie Y., Fan D. (2014). Long noncoding RNA MRUL promotes ABCB1 expression in multidrug-resistant gastric cancer cell sublines. Mol. Cell. Biol..

[240] Dong S., Qu X., Li W., Zhong X., Li P., Yang S., Chen X., Shao M., Zhang L. (2015). The long non-coding RNA, GAS5, enhances gefitinib-induced cell death in innate EGFR tyrosine kinase inhibitor-resistant lung adenocarcinoma cells with wide-type EGFR via downregulation of the IGF-1R expression. J. Hematol. Oncol..

[241] Cheng N., Cai W., Ren S., Li X., Wang Q., Pan H., Zhao M., Li J., Zhang Y., Zhao C. (2015). Long non-coding RNA UCA1 induces non-T790M acquired resistance to EGFR-TKIs by activating the AKT/mTOR pathway in EGFR-mutant non-small cell lung cancer. Oncotarget.

[242] Pan J., Li X., Wu W., Xue M., Hou H., Zhai W., Chen W. (2016). Long non-coding RNA UCA1 promotes cisplatin/gemcitabine resistance through CREB modulating miR-196a-5p in bladder cancer cells. Cancer Lett..

[243] Fan Y., Shen B., Tan M., Mu X., Qin Y., Zhang F., Liu Y. (2014). Long non-coding RNA UCA1 increases chemoresistance of bladder cancer cells by regulating Wnt signaling. FEBS J..

[244] He D.-X., Zhang G.-Y., Gu X.-T., Mao A.-Q., Lu C.-X., Jin J., Liu D.-Q., Ma X. (2016). Genome-wide profiling of long non-coding RNA expression patterns in anthracycline-resistant breast cancer cells. Int. J. Oncol..

[245] Jiang M., Huang O., Xie Z., Wu S., Zhang X., Shen A., Liu H., Chen X., Wu J., Lou Y. (2014). A novel long non-coding RNA-ARA: Adriamycin resistance-associated. Biochem. Pharmacol..

[246] Shi S.-J., Wang L.-J., Yu B., Li Y.-H., Jin Y., Bai X.-Z. (2015). LncRNA-ATB promotes trastuzumab resistance and invasion-metastasis cascade in breast cancer. Oncotarget.

[247] Li W., Zhai L., Wang H., Liu C., Zhang J., Chen W., Wei Q. (2016). Downregulation of LncRNA GAS5 causes trastuzumab resistance in breast cancer. Oncotarget.

[248] Schouten P.C., Vollebergh M.A., Opdam M., Jonkers M., Loden M., Wesseling J., Hauptmann M., Linn S.C. (2016). High XIST and Low 53BP1 Expression Predict Poor Outcome after High-Dose Alkylating Chemotherapy in Patients with a BRCA1-like Breast Cancer. Mol. Cancer Ther..

[249] Redis R.S., Sieuwerts A.M., Look M.P., Tudoran O., Ivan C., Spizzo R., Zhang X., de Weerd V., Shimizu M., Ling H. (2013). CCAT2, a novel long non-coding RNA in breast cancer: Expression study and clinical correlations. Oncotarget.

[250] Lee H., Kim C., Ku J.-L., Kim W., Yoon S.K., Kuh H.-J., Lee J.-H., Nam S. W., Lee E.K. (2014). A long non-coding RNA snaR contributes to 5-fluorouracil resistance in human colon cancer cells. Mol. Cells.

[251] Yue B., Cai D., Liu C., Fang C., Yan D. (2016). Linc00152 Functions as a Competing Endogenous RNA to Confer Oxaliplatin Resistance and Holds Prognostic Values in Colon Cancer. Mol. Ther. J. Am. Soc. Gene Ther..

[252] Li Y., Huang S., Li Y., Zhang W., He K., Zhao M., Lin H., Li D., Zhang H., Zheng Z. (2016). Decreased expression of LncRNA SLC25A25-AS1 promotes proliferation, chemoresistance, and EMT in colorectal cancer cells. Tumour Biol..

[253] Hang Q., Sun R., Jiang C., Li Y. (2015). Notch 1 promotes cisplatin-resistant gastric cancer formation by upregulating lncRNA AK022798 expression. Anticancer. Drugs.

[254] Zhang X., Bu P., Liu L., Zhang X., Li J. (2015). Overexpression of long non-coding RNA PVT1 in gastric cancer cells promotes the development of multidrug resistance. Biochem. Biophys. Res. Commun..

[255] Takahashi K., Yan I.K., Kogure T., Haga H., Patel T. (2014). Extracellular vesicle-mediated transfer of long non-coding RNA ROR modulates chemosensitivity in human hepatocellular cancer. FEBS Open Bio.

[256] Chen J., Zhang K., Song H., Wang R., Chu X., Chen L. (2016). Long noncoding RNA CCAT1 acts as an oncogene and promotes chemoresistance in docetaxel-resistant lung adenocarcinoma cells. Oncotarget.

[257] Yang Y., Li H., Hou S., Hu B., Liu J., Wang J. (2013). The noncoding RNA expression profile and the effect of lncRNA AK126698 on cisplatin resistance in non-small-cell lung cancer cell. PLoS ONE.

[258] Liu J., Wan L., Lu K., Sun M., Pan X., Zhang P., Lu B., Liu G., Wang Z. (2015). The long noncoding RNA MEG3 contributes to cisplatin resistance of human lung adenocarcinoma. PLoS ONE.

[259] Mourtada-Maarabouni M., Williams G.T. (2014). Role of GAS5 noncoding RNA in mediating the effects of rapamycin and its analogues on mantle cell lymphoma cells. Clin. Lymphoma Myeloma Leuk..

[260] Ren S., Li G., Liu C., Cai T., Su Z., Wei M., She L., Tian Y., Qiu Y., Zhang X. (2016). Next generation deep sequencing identified a novel lncRNA n375709 associated with paclitaxel resistance in nasopharyngeal carcinoma. Oncol. Rep..

[261] Li L., Gu M., You B., Shi S., Shan Y., Bao L., You Y. (2016). Long non-coding RNA ROR promotes proliferation, migration and chemoresistance of nasopharyngeal carcinoma. Cancer Sci..

[262] Jiang L., Wang W., Li G., Sun C., Ren Z., Sheng H., Gao H., Wang C., Yu H. (2016). High TUG1 expression is associated with chemotherapy resistance and poor prognosis in esophageal squamous cell carcinoma. Cancer Chemother. Pharmacol..

[263] Wang Y., Zhang L., Zheng X., Zhong W., Tian X., Yin B., Tian K., Zhang W. (2016). Long non-coding RNA LINC00161 sensitises osteosarcoma cells to cisplatin-induced apoptosis by regulating the miR-645-IFIT2 axis. Cancer Lett..

[264] Zhang C.-L., Zhu K.-P., Shen G.-Q., Zhu Z.-S. (2016). A long non-coding RNA contributes to doxorubicin resistance of osteosarcoma. Tumour Biol..

[265] Zhu K.-P., Zhang C.-L., Shen G.-Q., Zhu Z.-S. (2015). Long noncoding RNA expression profiles of the doxorubicin-resistant human osteosarcoma cell line MG63/DXR and its parental cell line MG63 as ascertained by microarray analysis. Int. J. Clin. Exp. Pathol..

[266] Liu E., Liu Z., Zhou Y., Mi R., Wang D. (2015). Overexpression of long non-coding RNA PVT1 in ovarian cancer cells promotes cisplatin resistance by regulating apoptotic pathways. Int. J. Clin. Exp. Med..

[267] Zhang L., Cao X., Zhang L., Zhang X., Sheng H., Tao K. (2016). UCA1 overexpression predicts clinical outcome of patients with ovarian cancer receiving adjuvant chemotherapy. Cancer Chemother. Pharmacol..

[268] Wu D.I., Wang T., Ren C., Liu L., Kong D., Jin X., Li X., Zhang G. (2016). Downregulation of BC200 in ovarian cancer contributes to cancer cell proliferation and chemoresistance to carboplatin. Oncol. Lett..

[269] Li Z., Zhao X., Zhou Y., Liu Y., Zhou Q., Ye H., Wang Y., Zeng J., Song Y., Gao W. (2015). The long non-coding RNA HOTTIP promotes progression and gemcitabine resistance by regulating HOXA13 in pancreatic cancer. J. Transl. Med..

[270] You L., Chang D., Du H.-Z., Zhao Y.-P. (2011). Genome-wide screen identifies PVT1 as a regulator of Gemcitabine sensitivity in human pancreatic cancer cells. Biochem. Biophys. Res. Commun..

[271] Jiao F., Hu H., Han T., Yuan C., Wang L., Jin Z., Guo Z., Wang L. (2015). Long noncoding RNA MALAT-1 enhances stem cell-like phenotypes in pancreatic cancer cells. Int. J. Mol. Sci..

[272] Yacqub-Usman K., Pickard M.R., Williams G.T. (2015). Reciprocal regulation of GAS5 lncRNA levels and mTOR inhibitor action in prostate cancer cells. Prostate.

[273] Xu C.-G., Yang M.-F., Ren Y.-Q., Wu C.-H., Wang L.-Q. (2016). Exosomes mediated transfer of lncRNA UCA1 results in increased tamoxifen resistance in breast cancer cells. Eur. Rev. Med. Pharmacol. Sci..

